# BET inhibitor suppresses melanoma progression *via* the noncanonical NF-κB/SPP1 pathway

**DOI:** 10.7150/thno.47432

**Published:** 2020-09-15

**Authors:** Guangtong Deng, Furong Zeng, Juan Su, Shuang Zhao, Rui Hu, Wu Zhu, Shuo Hu, Xiang Chen, Mingzhu Yin

**Affiliations:** 1 Department of Dermatology, Hunan Engineering Research Center of Skin Health and Disease, Hunan Key Laboratory of Skin Cancer and Psoriasis, Xiangya Hospital, Central South University, Changsha, Hunan 410008, China.; 2 National Clinical Research Center for Geriatric Disorders, Xiangya Hospital, Central South University, Changsha, Hunan 410008, China.; 3 Department of PET Center, Xiangya Hospital, Central South University, Changsha, 410008, China.

**Keywords:** BET inhibitor, SPP1, noncanonical NF-κB pathway, melanoma.

## Abstract

**Background:** Bromodomain and extra-terminal domain (BET) inhibitors have shown profound efficacy against hematologic malignancies and solid tumors in preclinical studies. However, the underlying molecular mechanism in melanoma is not well understood. Here we identified secreted phosphoprotein 1 (SPP1) as a melanoma driver and a crucial target of BET inhibitors in melanoma. **Methods:** Bioinformatics analysis and meta-analysis were used to evaluate the SPP1 expression in normal tissues, primary melanoma, and metastatic melanoma. Real-time PCR (RT-PCR) and Western blotting were employed to quantify SPP1 expression in melanoma cells and tissues. Cell proliferation, wound healing, and Transwell assays were carried out to evaluate the effects of SPP1 and BET inhibitors in melanoma cells *in vitro*. A xenograft mouse model was used to investigate the effect of SPP1 and BET inhibitors on melanoma *in vivo*. Chromatin immunoprecipitation (ChIP) assay was performed to evaluate the regulatory mechanism of BET inhibitors on SPP1. **Results:** SPP1 was identified as a melanoma driver by bioinformatics analysis, and meta-analysis determined it to be a diagnostic and prognostic biomarker for melanoma. SPP1 overexpression was associated with poor melanoma prognosis, and silencing SPP1 suppressed melanoma cell proliferation, migration, and invasion. Through a pilot drug screen, we identified BET inhibitors as ideal therapeutic agents that suppressed SPP1 expression. Also, SPP1 overexpression could partially reverse the suppressive effect of BET inhibitors on melanoma. We further demonstrated that bromodomain-containing 4 (BRD4) regulated SPP1 expression. Notably, BRD4 did not bind directly to the SPP1 promoter but regulated SPP1 expression through NFKB2. Silencing of NFKB2 resembled the phenotype of BET inhibitors treatment and SPP1 silencing in melanoma. **Conclusion:** Our findings highlight SPP1 as an essential target of BET inhibitors and provide a novel mechanism by which BET inhibitors suppress melanoma progression *via* the noncanonical NF-κB/SPP1 pathway.

## Introduction

Melanoma is one of the most lethal cutaneous tumors with increasing incidence and mortality in recent decades due to its invasiveness and metastasis 1. Targeted therapies, such as B-Raf proto-oncogene (BRAF) and MAP kinase-ERK kinase (MEK) inhibitors, and immunotherapy, such as anti-CTLA-4 and anti-PD-1/PD-L1, have been employed in the clinic with good response in some patients 2. However, limited indications, quick resistance development, and toxicity of these treatments highlight the need for developing new therapeutic strategies 3.

Targeting chromatin regulators has become an attractive antitumor strategy due to the widely observed epigenetic dysregulation in tumors. Different categories of epigenetic drugs have been approved to treat various cancers, such as inhibitors of DNA methyltransferases, histone demethylases, and histone methyltransferase enhancer of zeste 2 polycomb repressive complex 2 subunit (EZH2). Recently, the tumor promotion functions of bromodomain and extra-terminal domain (BET) proteins, including BRD2, BRD3, BRD4, and BRDT, which bind to acetylated histones, have attracted great attention 4. Several small-molecule BET inhibitors, including NHWD-870 by our team, have been developed that have shown profound efficacy against hematologic and solid tumors in preclinical studies 5, 6. A previous study demonstrated that BRD4 was commonly overexpressed in melanoma and facilitated recruitment of the transcription elongation factor to drive melanoma progression 7. MYC is a well-established BRD4 target; however, the re-introduction of MYC failed to rescue the growth suppression by BET inhibitors in melanoma, even with the combination of the cyclin-dependent kinase (CKD) inhibitor therapy and p21 knockdown 7. These findings suggested the existence of other targets of BET proteins that are critical for melanoma progression and are responsible for BET inhibitor-mediated growth arrest.

Gene expression profiling and bioinformatics analyses have been used to explore tumor progression mechanisms and to identify prognostic biomarkers 8, 9. Our study mined multiple gene expression profiles from normal skin, primary and metastatic melanoma, and identified secreted phosphoprotein 1 (SPP1) as a potential melanoma driver. SPP1, an integrin-binding phosphorylated glycoprotein, has been reported to be differentially expressed in a variety of cancer cells 10. SPP1 plays an important role in several tumor-associated processes, including proliferation, invasion, migration, angiogenesis, and metastasis 11-13. However, the role of SPP1 in melanoma and its regulation have not been studied well.

In this study, we found that SPP1 was highly expressed in human melanoma and enhanced cell proliferation, migration, and invasion. Overexpression of SPP1 predicted poor prognosis in melanoma. Through drug screening, we found that BET inhibitors prevented SPP1 expression in a dose-dependent manner, and re-introduction of SPP1 could partially reverse the growth inhibition of BET inhibitors in melanoma. We further demonstrated that the inhibition of BRD4 suppressed SPP1 expression through NFKB2, a member of the noncanonical nuclear factor kappa B (NF-κB) pathway. Also, siRNA-mediated down-regulation of NFKB2 resulted in reduced melanoma cell proliferation, migration, and invasion. These findings identified the BRD4-NFKB2-SPP1 axis as a new oncogenic pathway in melanoma progression, and that SPP1 may be an ideal target of BET inhibitors for melanoma treatment.

## Materials and methods

### Microarray data

GSE15605 and GSE46517 profiles were selected from the Gene Expression Omnibus (GEO) database (http://www.ncbi.nlm.nih.gov/geo/). The GSE15605 dataset, including 16 normal skin, 46 primary melanoma, and 12 metastatic melanoma samples, is based on the GPL570 platform (HG-U133_Plus_2, Affymetrix Human Genome U133 Plus 2.0 Array). The GSE46517 dataset is based on the GPL96 platform (HG-U133A Affymetrix Human Genome U133A Array) and consisted of 7 normal skin, 31 primary melanoma, and 73 metastatic melanoma samples. The GSE8401 dataset was used to compare SPP1 expression in primary versus metastatic melanoma based on the GPL96 platform and included 31 primary melanoma and 62 metastatic melanoma samples. The Cancer Genome Atlas (TCGA) dataset of melanoma was used to assess the SPP1 expression in primary melanoma versus metastatic melanoma, which was downloaded from the TCGA data portal (https://portal.gdc.cancer.gov/).

### Oncomine, GEPIA, and HPA databases

Oncomine is a tumor microarray database and an integrated web-based data-mining platform (https://www.oncomine.org/resource/main.html) 14, which was used to validate SPP1 expression between normal skin and melanoma samples at the transcriptional level. Gene expression profiling interactive analysis (GEPIA) is a web server for cancer and normal gene expression profiling and interactive analyses 15 and integrates TCGA and GTEx data (http://gepia.cancer-pku.cn/). GEPIA was used to explore the SPP1 expression between different subtypes of melanoma (WT and BRAF, NF1, and NRAS mutants) and normal skin, as well as SPP1 expression in different pathological grades of melanoma. The Human Protein Atlas (HPA) maps all human proteins in cells, tissues, and organs (https://www.proteinatlas.org/). SPP1 protein level was evaluated based on its immunohistochemistry (IHC) staining between melanoma and normal skin in HPA using antibody CAB002212. Genes were considered differentially expressed if the |log (fold change) | >1 and the *t*-test *P*-value < 0.05.

### Patients characteristics and RNA-sequencing (RNA-seq)

Snap-frozen melanoma samples were obtained from 19 patients, who underwent surgery at Xiangya Hospital of Central South University from August 8^th^, 2013 to August 15^th^, 2014. Among them, matched specimens of melanoma and adjacent non-tumor tissue from 11 cases were collected. The clinical outcomes were monitored up to June 30th, 2019, the last day of follow-up. To explore the relationship between SPP1 expression and overall survival and clinicopathological variables, RNA-seq analysis was performed at the Genergy Biotechnology Company (http://genergy.bioon.com.cn/, Shanghai, China). Total RNA was extracted from snap-frozen melanoma and paired adjacent normal tissues using the RNeasy Mini Kit (QIAGEN, Germantown, USA) according to the manual instructions. Library construction was conducted with TruSeq® RNA LT Sample Prep Kit v2 (Illumina, San Diego, USA). Sequencing was performed on a Hiseq3000 SBS platform after library construction. Ethical approval for the study was obtained from the Ethics Committee of Central South University and written informed consent was obtained from each patient.

### Meta-analysis

GD and FZ independently searched PubMed, Embase, and Web of Science until September 24^th^, 2019. Details of the search strategies are described in **Table S1**. Studies reporting the SPP1 protein level in melanoma patients based on IHC staining or enzyme-linked immunosorbent assay (ELISA) were selected if they met the following inclusion criteria: (1) studies comparing SPP1 expression among normal skin, primary melanoma, and metastatic melanoma; or (2) studies including melanoma survival information based on SPP1 expression. If there were two or more studies from the same authors or institutions, only the study with the largest sample size was chosen. Studies were excluded if they were not available or did not fulfill the inclusion criteria. The records from the initial search were scanned by GD and FZ to exclude any duplicate or irrelevant studies. Any discrepancies were resolved by discussion. Study quality was assessed using the Newcastle Ottawa Scale (NOS), and studies above 6 stars were considered to be of high quality. Statistical heterogeneity was evaluated by I^2^ and P values. A fixed-effects model was adopted if there was no evidence of significant heterogeneity (I^2^ ≤ 50% and P ≥ 0.1); otherwise, the random-effects model was used. Sensitivity analysis and publication bias evaluation were conducted through influence analysis and the Egger test.

### Cell culture, lentiviral infection, and transfection

The melanoma cell lines A375 and SK-MEL-28 were purchased from ATCC (Manassas, USA) and cultured in high glucose Dulbecco's Modified Eagle's Medium (DMEM) (BI, Israel) supplemented with 10% fetal bovine serum (FBS) (BI, Israel) and antibiotics. All cells were incubated at 37℃ in humidified air with 5% CO_2_. The pLKD-CMV-EGFP-2A-Puro-U6-shRNA-SPP1 and pLKD-CMV-EGFP-2A-Puro plasmids were purchased from Obio Technology Corp., Ltd. (Shanghai, China). For the generation of stable SPP1 knockdown cells, shRNA, pMD2.G (Addgene, Cambridge, USA) and psPAX2 (Addgene, Cambridge, USA) were co-transfected into HEK-293T cells by using TurboFect (Thermo Fisher Scientific, Waltham, USA) according to the manufacturer's instructions. Virus-containing cell culture supernatant was collected 48 and 72 hours after transfection and added to A375 or SK-MEL-28 cells. Transduced cells were used for experiments after puromycin (Thermo Fisher Scientific, Waltham, USA) selection for 48 hours. RT-PCR and Western blotting were employed to verify the transfection efficiency. Interfering sequences were: shSPP1#1 (GCTACAGACGAGGACATCA), shSPP1#2 (CCGTGGGAAGGACAGTTAT). Transfection of siRNA (Ruibobio, Guangzhou, China) was performed according to manufacturer's instructions. Non-targeting siRNA was used as a control. Briefly, cells were seeded at 70% confluence in 6-well or 12-well culture plates, then transfected with siRNAs by using TurboFect and incubated for 48 hours. The concentration of siRNAs was optimized based on dose-response studies. Interfering sequences are shown in **Table S2**.

### Real-time reverse transcription PCR (RT-PCR)

Total RNA was extracted from cell lines using TRIpure reagent (BioTek, Winooski, USA), and reverse-transcribed to cDNA using the HiScript Q RT SuperMix from the qPCR kit (Vazyme, Nanjing, China). RT-PCR was performed using specific primers and Applied Biosystems QuantStudio™ 3 Real-Time PCR System (Thermo Fisher Scientific, Waltham, USA) with SYBR Green Master Mix (CWBIO, Jiangsu, China). Target gene expression values were normalized to human GAPDH. The primers used for RT-PCR are shown in **Table S3**.

### Western blotting

Cells were harvested and lysed on ice with RIPA buffer. The protein samples were analyzed on 10% or 12% SDS-PAGE and transferred onto a polyvinylidene fluoride (PVDF) membrane. The membranes were blocked with 5% non-fat milk for one hour at room temperature, washed three times in TBST, and incubated with the primary antibody overnight at 4 °C. The membranes were washed three times with TBST and incubated with horseradish peroxidase secondary antibodies (ABclonal, Wuhan, China) for one hour at room temperature. The bands were detected using Western ECL Blotting Substrates according to the manufacturer's protocols. The following primary antibodies were used: anti-SPP1 (ProMab, Richmond, USA); anti-BRD4 (Abcam, Cambridge, USA); anti-NFKB2 (ProMab, Richmond, USA); anti-MMP2 (Proteintech, Wuhan, China); anti-Actin (Santa Cruz, Dallas, USA).

### Cell Counting Kit-8 (CCK-8) assay

Cell proliferation was measured by the Cell Counting Kit-8 (CCK-8) assay (Bimake, Houston, USA). In brief, cells were seeded (10^3^/well) in 96-well plates overnight at 37 °C in 100 μl media containing 10% FBS to allow cell attachment. Ten microliters CCK-8 solution was added to each well for 2 hours of incubation. The absorbance values were detected at a wavelength of 450 nm using a 96-well microplate reader (BioTek, Winooski, USA).

### Scratch-wound healing assay

Cell migration was determined by wound healing assay. Cells were seeded in 6-well plates and scraped straightly using a P-200 pipette tip when they reached 95% confluence, then washed with phosphate-buffered saline (PBS) three times to remove the floating cells. Six random images were taken immediately by inverted microscope at 0, 24, and 48 hours after scraping. The wound surface area was quantified by Image J software.

### Transwell invasion assay

Transwells with 8 μm pores (Corning, Corning, USA) were precoated with Matrigel (1:7 in DMEM, Corning, Corning, USA). Cells were seeded at 5×10^4^/well in serum-free medium in the upper chambers after overnight starvation. The cells were then allowed to invade the lower chamber containing medium supplemented with 10% FBS. The inserts were then fixed with 4% paraformaldehyde for 15 min and stained with 0.5% crystal violet for 15 min. Migrated cells from five random fields were photographed by an inverted microscope and manually counted.

### Animal study

Pathogen-free BALB/c nude female mice and NOD-SCID-gamma (NSG) mice (6-8 weeks old) were obtained from the Department of Laboratory Animals, Central South University. All studies were conducted according to the experimental protocol approved by the Ethical Review of Experimental Animals at Central South University. shSPP1, shCtrl and WT human A375 cells (10^6^) were injected subcutaneously into the right flank of each BALB/c nude mouse or NSG mouse in a volume of 100 μl PBS. When the tumor reached 100mm^3^, NHWD-870 (1 mg/kg) or vehicle (0.5% methylcellulose + 0.1% Tween 80) was orally given to BALB/c nude mouse once a day for five successive days and then two days off. Tumor size and body weight were recorded twice per week. Tumor size was determined by Vernier caliper measurement and calculated as ([length×width^2^]/2). The animals were sacrificed on day 21, and tumors were photographed.

### ChIP-seq and ChIP-qPCR

Chromatin immunoprecipitation (ChIP) was performed by Acegen company (http://www.sz-acegen.com/, Shenzhen, China). Briefly, A375 cells were collected and cross-linked with 1% formaldehyde and then sonicated by a Bioruptor to generate chromatin fragments between 100 and 750 bp. Sonicated samples were then immunoprecipitated using the BRD4 antibody. DNA library was prepared using Acegen DNA Library Prep Kit from Illumina, then twelve-cycle PCR amplified, cleaned up, and analyzed by Agilent 2100 Bioanalyzer and finally sequenced on the Illumina platform. The purified chromatin templates were amplified using RT-PCR. The following primers were used to detect the binding of BRD4 to the promoter region of NFKB2. Forward primer: AGAGATCTCCCTGTCGCCTG, and reverse primer: TGCGGGAAAAGTGTCTCCTC.

### Statistical analyses

Bioinformatics analysis was conducted by using R software (https://www.r-project.org/). Meta-analysis calculations were performed by STATA software (Version 12.0; STATA Corporation, College Station, TX, USA). All experiments were performed in triplicate. The data were analyzed using GraphPad Prism 5 (GraphPad Software, La Jolla, CA). The values were expressed as mean ± standard deviation, computed for each group, and analyzed using the Student *t*-test. We used the Kaplan-Meier method to generate survival curves and a log-rank test to determine whether gene levels were significantly associated with overall patient survival. A p-value of <0.05 was considered to indicate a statistically significant difference.

## Results

### Identification of SPP1 as a potential melanoma driver

**Figure 1:**
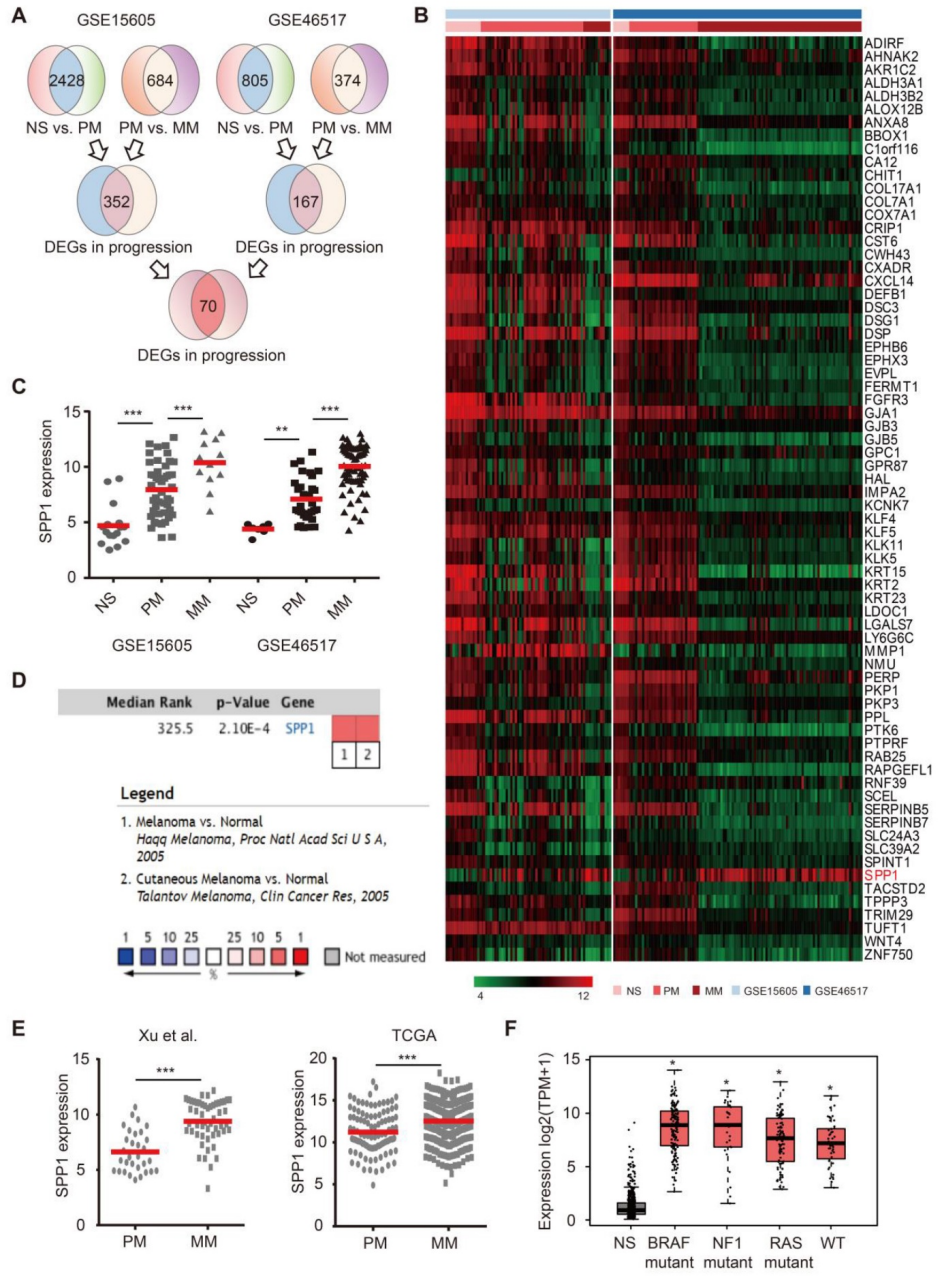
** Identification of secreted phosphoprotein 1 (SPP1) as a potential melanoma driver. (A)** Schematic flowchart of gene selection in GSE15605 and GSE46517 from GEO database. **(B)** Heatmap of 70 genes that differentially expressed among normal skin (NS), primary (PM), and metastatic (MM) melanoma in both GSE15605 and GSE46517. **(C)** SPP1 expression in NS, PM, and MM from GSE15605 and GSE46517. The number of sorts: *N* (NS) = 16, *N* (PM) = 46, and *N* (MM) = 12 in GSE15605; *N* (NS) = 7, *N* (PM) = 31, and *N* (MM) = 73 in GSE46517. **(D)** SPP1 expression in NS and melanoma tissue from Oncomine database. **(E)** SPP1 expression in PM and MM from Xu *et al.*, 2018(GSE8401) and TCGA. The number of sorts: *N* (PM) = 31 and* N* (MM) = 52 from Xu *et al.*, 2018(GSE8401); *N* (PM) = 103 and *N* (MM) = 369 from TCGA. **(F)** SPP1 expression in three mutational signatures (BRAF, NF1, and RAS) and wild types (WT) of melanoma based on GEPIA (Gene Expression Profiling Interactive Analysis). The number of sorts: *N* (NS) = 558, *N* (BRAF) = 147, *N* (NF1) = 27, *N* (RAS) = 91 and *N* (WT) = 47. Error bars represent standard deviation (SD). *P*-values were calculated using unpaired two-tailed Student's t test. *, *P* < 0.05; **, *P* < 0.01; ***, *P* < 0.001.

To identify key melanoma drivers, we mined two public datasets. The first cohort (GSE15605) contained RNA-seq results from 16 normal skin, 46 primary melanoma, and 12 metastatic melanoma samples. The second cohort (GSE46517) consisted of 7 normal skin, 31 primary melanoma, and 73 metastatic melanoma samples. After normalization** (Figure S1A)**, 70 differentially expressed genes (DEGs) were selected among normal skin, primary melanoma, and metastatic melanoma based on our selection strategy **(Figure 1A-B, Figure S1B)**. There were 67 DEGs with high expression in normal skin, moderate expression in primary melanoma, and low expression in metastatic melanoma **(Figure S1C)**. However, SPP1 was identified as the only potential melanoma driver due to its high expression in metastatic melanoma, moderate expression in primary melanoma, and low expression in normal skin **(Figure 1C and Figure S1D)**. The Oncomine database confirmed that SPP1 was overexpressed in melanoma compared to normal skin** (Figure 1D)**. Moreover, melanoma samples from two independent cohorts (TCGA and GSE8401) also demonstrated significant up-regulation of SPP1 in metastatic versus primary melanoma samples **(Figure 1E)**. We also found that SPP1 overexpression in melanoma was independent of key melanoma mutations** (Figure 1F)**. Overall, we identified SPP1 as a potential melanoma driver through bioinformatics analysis.

### SPP1 acts as a biomarker for diagnosis and progression of melanoma in meta-analysis

**Figure 2:**
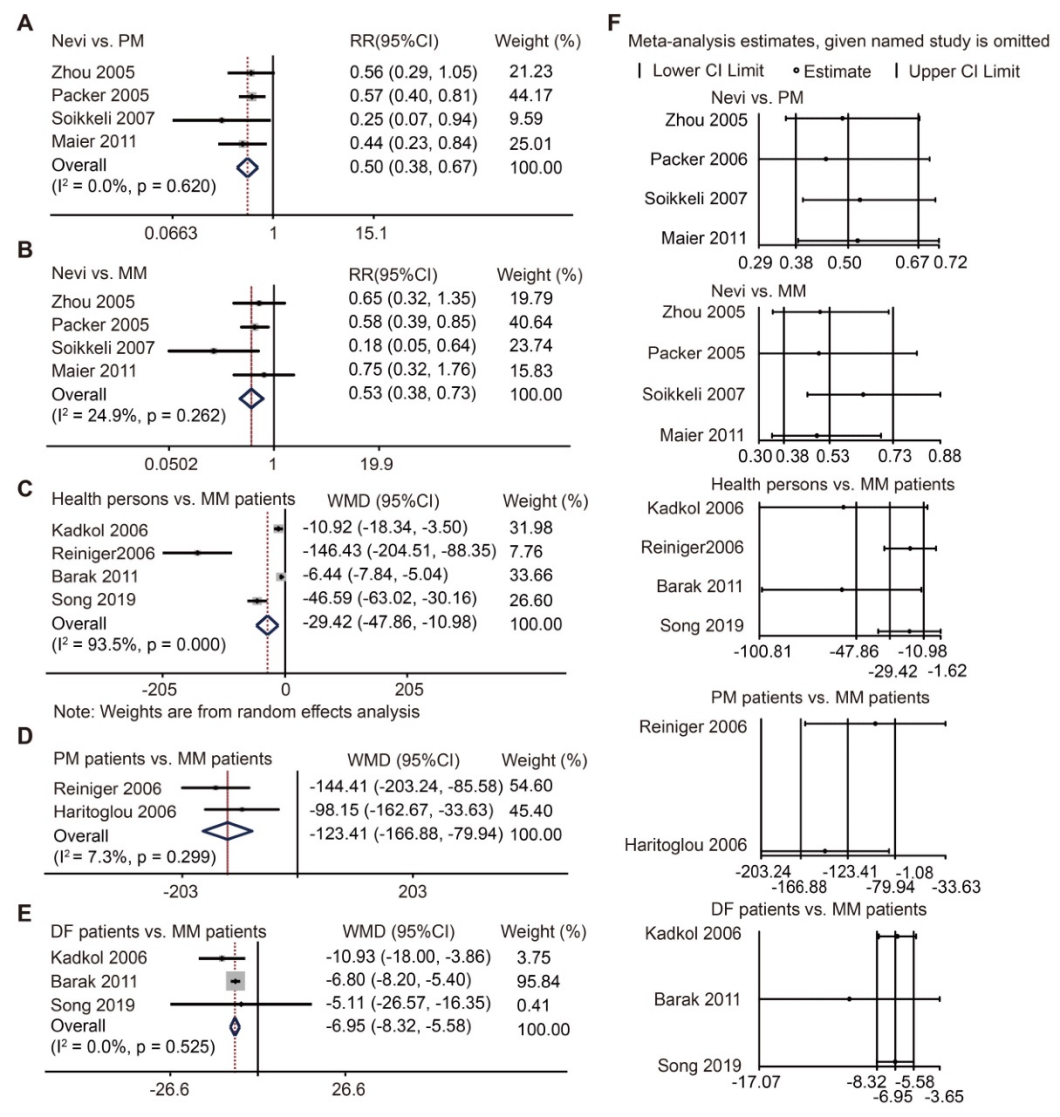
** SPP1 acts as a biomarker for diagnosis and progression of melanoma through meta-analysis. (A-B)** Forest plot of SPP1 expression between nevi and PM** (A)**, between nevi and MM** (B)** based on immunohistochemistry staining. **(C-E)** Forest plot of SPP1 expression between health persons and MM** (C)**, between PM and MM patients **(D)**, between patients who were disease free (DF) for at least five years and MM patients **(E)** based on enzyme linked immunosorbent assay.** (F)** Sensitivity analysis.

SPP1 protein level has been studied previously but was not comprehensively defined. Here, we conducted a meta-analysis to explore its role in melanoma. Through our search strategy and selection flowchart **(Figure S2)**, twelve cohort studies were ultimately included in our analysis 16-27. The characteristics of these eligible studies were comparable** (Table S4).** Study quality was assessed using the Newcastle Ottawa Scale (NOS) **(Table S5)**. There were seven studies using IHC staining to evaluate SPP1 expression 17, 18, 20, 22, 24, 26, 27. Pooled data showed more SPP1-positive staining in primary and metastatic melanoma than in nevi **(Figure 2A-B)**, though there was no significant difference in SPP1-positive staining between primary and metastatic melanoma **(Figure S3A-B)**. Given that SPP1 is a secreted protein, we also included five studies that used ELISA to detect SPP1 concentration in patient serum samples 16, 19, 21, 23, 25. The serum SPP1 concentration was higher in metastatic melanoma patients than healthy individuals, primary melanoma patients, and those who were disease-free for at least five years after treatment **(Figure 2C-E)**. Moreover, one study each on SPP1 concentration between healthy persons and primary melanoma patients 23, melanoma-specific survival and overall survival based on ELISA 28, and recurrence-free survival and disease-specific survival based on IHC staining 29, were not included in the meta-analysis due to their inadequate data. Sensitivity analysis did not change the conclusion and there was no publication bias for these comparisons **(Figure 2F, Table S6)**. Our findings suggested that SPP1 could act as a biomarker for the diagnosis and progression of melanoma.

### Up-regulation of SPP1 predicts poor prognosis in human melanoma

**Figure 3:**
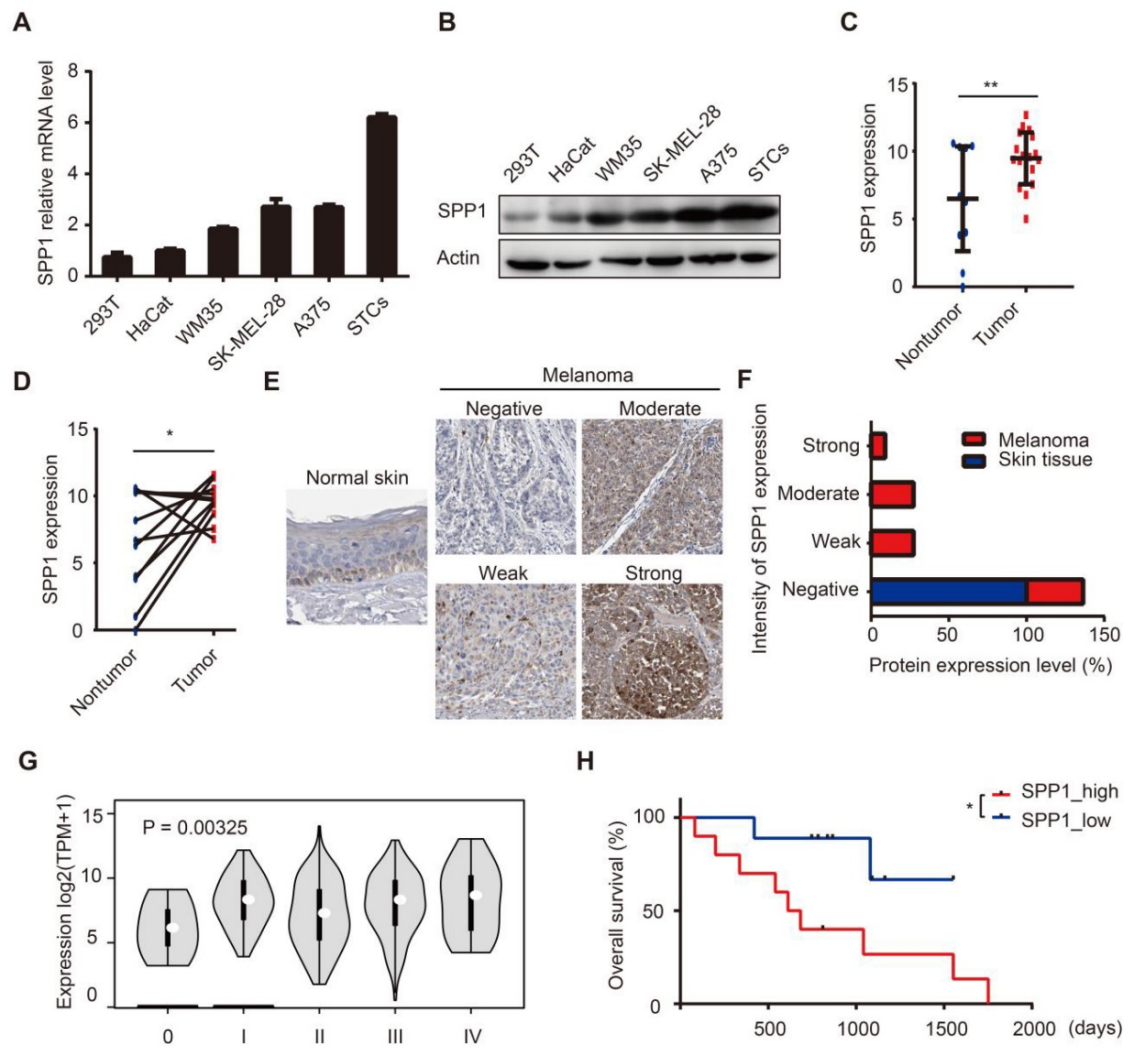
** Up-regulation of SPP1 predicts poor prognosis in human melanoma. (A-B)** Relative expression levels of SPP1 in HEK-293T, HaCat cells, melanoma cell lines, and patients-derived melanoma short-term cultures (STCs) quantified by RT-PCR **(A)** and western blotting **(B)**. **(C-D)** Quantification by RNA-seq of SPP1 expression between melanoma and melanoma-adjacent tissue (*N* (nontumor) = 11; *N* (tumor) = 19)** (C)**, between melanoma and paired melanoma-adjacent tissue from Xiangya melanoma cohort (*N* = 11)** (D)**. **(E-F)** The representative protein expression of SPP1** (E)** and percent distribution** (F)** between normal skin and melanoma tissues in The Human Protein Atlas (https://www.proteinatlas.org). **(G)** SPP1 expression grouped by pathologic grades of melanoma from GEPIA.** (H)** Kaplan-Meier survival analysis of high and low SPP1 expression groups in Xiangya melanoma cohort. Error bars represent SD. *, *P* < 0.05; **, *P* < 0.01.

We assessed SPP1 expression in HEK-293T, HaCaT, and a panel of melanoma cell lines. SPP1 was highly expressed in melanoma cell lines and in patient-derived melanoma short-term cultures **(Figure 3A-B)**. Subsequently, we performed RNA-seq analysis on surgically removed tumor and tumor-adjacent tissues from melanoma patients in Xiangya hospital. Our data demonstrated up-regulated SPP1 expression in melanoma tissues compared with adjacent tissues **(Figure 3C-D)**. IHC staining showed higher intensities of SPP1 in melanoma tissues than in tumor-adjacent normal tissues **(Figure 3E-F)**. More importantly, SPP1 expression was positively correlated with the pathologic grades of melanoma **(Figure 3G)**. To evaluate the correlation among SPP1 and clinicopathological variables, SPP1 expression in the Xiangya melanoma cohort was divided into the low expression and high expression groups. There was a significant association between elevated SPP1 expression and increasing Breslow thickness (P = 0.002) and unfavorable survival of melanoma patients **(Table S7, Figure 3H)**. To further explore the clinical value of SPP1 for melanoma prognosis, univariate and multivariate survival analyses were performed, suggesting that SPP1 expression was an independent risk factor for overall survival** (Table S8)**.

### SPP1 knockdown inhibits melanoma cell proliferation, migration, and invasion

**Figure 4:**
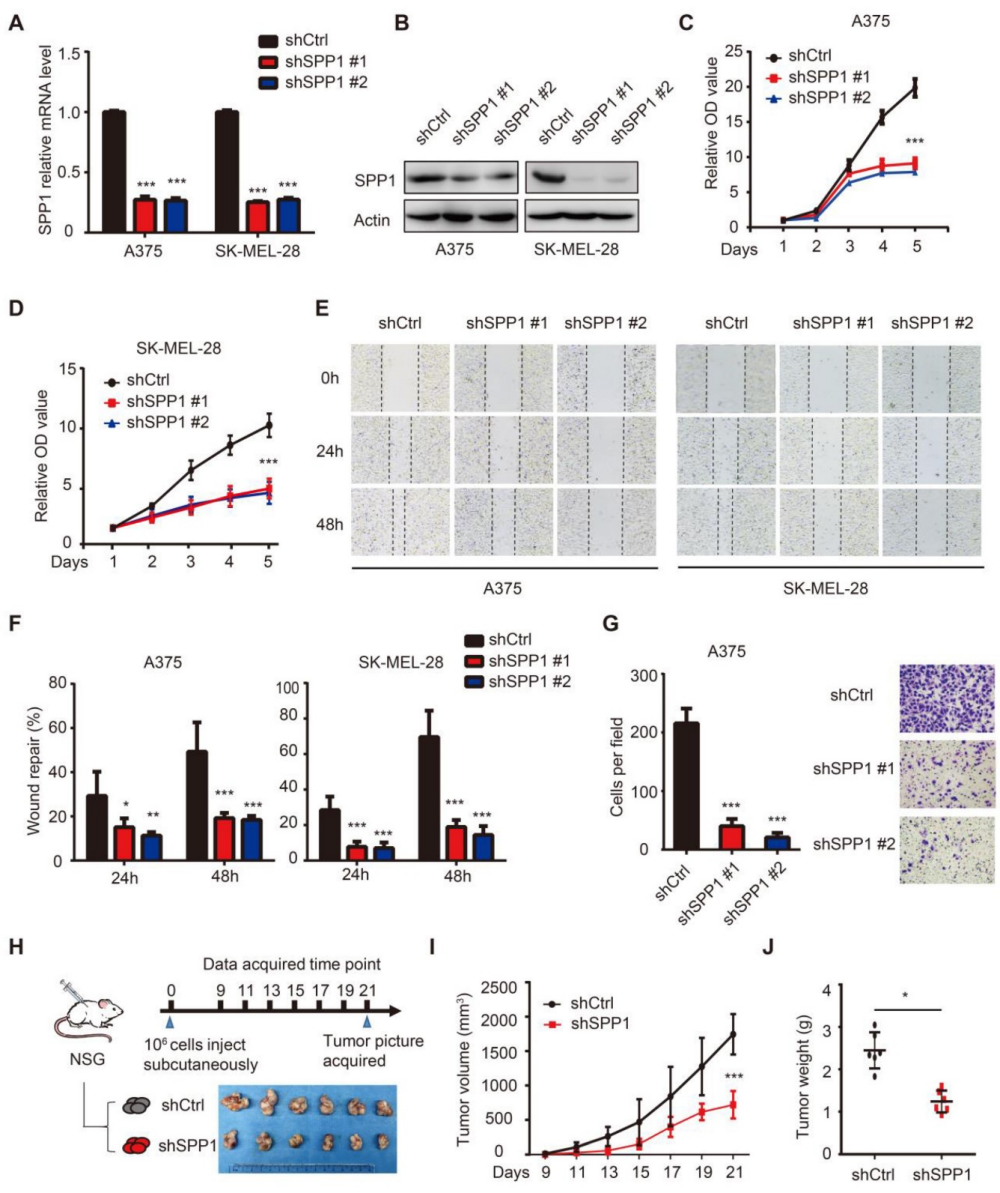
** Knockdown of SPP1 inhibits melanoma cell proliferation, migration and invasion. (A-B)** SPP1 knockdown efficiency in A375 and SK-MEL-28 cell lines stably transduced with control shRNA (shCtrl) or two SPP1 shRNAs (shSPP1 #1 and #2) quantified by RT-PCR **(A)** and western blotting** (B)**. **(C-D)** Cell proliferation of A375 **(C)** and SK-MEL-28** (D)** cells transduced with shCtrl, shSPP1 #1, and shSPP1 #2 were quantified by CCK-8 assay. **(E-F)** Scratch-wound healing assay of A375 and SK-MEL-28 cells transduced with shCtrl, shSPP1 #1, and shSPP1 #2. Six random areas were selected. Images (at 40X magnification) were taken at 0, 24, and 48 hours.** (G)** Invasiveness of A375 cells transduced with shCtrl, shSPP1 #1, and shSPP1 #2 were assessed by Transwell assays. Invaded cells were determined for 18 hours. Five random areas were selected. Images were taken at 200X magnification. **(H)** model pattern and picture of resected subcutaneous xenografted tumors. A375 cells (10^6^) were injected subcutaneously in the right flank of NSG mice. Tumors were resected and photographed at day 21 (N = 6 in each group). **(I)** Tumor volume were recorded every other day and calculated as ([length×width^2^]/2). **(J)** Tumor weights were recorded after tumor resection at day 21. All data were represented as mean ± SD of three independent experiments. *, *P* < 0.05; **, *P* < 0.01; ***, *P <* 0.001.

To assess the SPP1 function in melanoma, we stably suppressed SPP1 expression using two independent shRNAs in A375 and SK-MEL-28 melanoma cell lines **(Figure 4A-B)**. As shown in **Figure 4C-D,** SPP1 silencing markedly suppressed cell proliferation. Given that SPP1 was overexpressed in metastatic melanoma, wound healing and Transwell assays were performed to evaluate the role of SPP1 in mediating melanoma migration and invasion, respectively. Delayed wound repair was found after 24 and 48 hours in SPP1-silenced A375 and SK-MEL-28 cells **(Figure 4E-F)**. The number of invasive cells was also significantly decreased after SPP1 silencing in A375 cells** (Figure 4G)**. Next, we used a xenograft model in NSG mice to examine the effect of SPP1 knockdown on melanoma cell growth *in vivo*** (Figure 4H)**. Consistent with the *in vitro* data, SPP1 knockdown in A375 cells caused significant growth delay in xenografted melanoma tumors **(Figure 4I-J)**.

### BET inhibitors impede melanoma cell proliferation, migration, and invasion through SPP1

**Figure 5:**
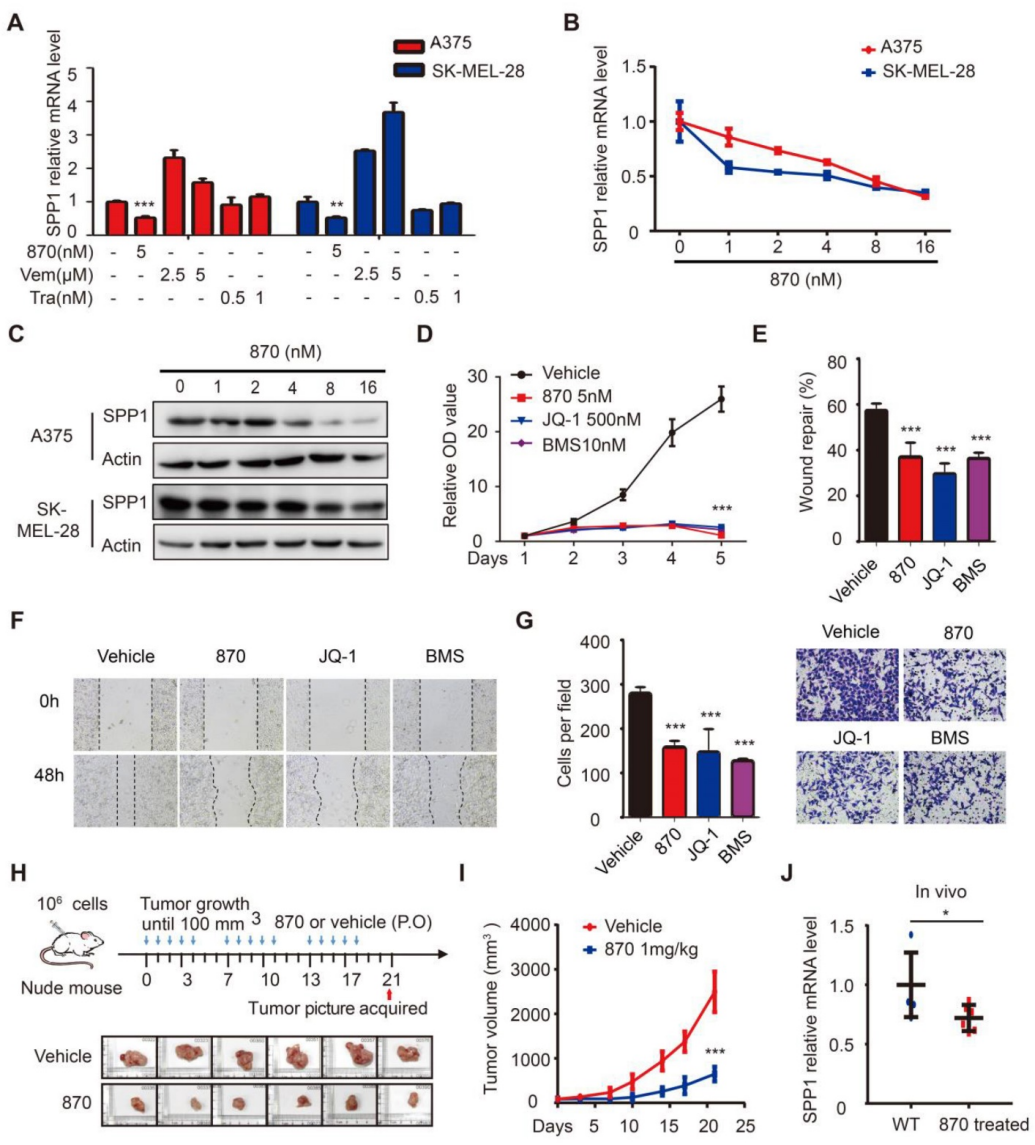
** BET inhibitors impede melanoma cell proliferation, migration, and invasion through SPP1. (A)** Quantification by RT-PCR of SPP1 expression in A375 and SK-MEL-28 cells after treatment with BET inhibitor (NHWD-870), BRAF inhibitor (Vemurafenib) or MEK inhibitor (Trametinib) for 24 hours. **(B-C)** SPP1 expression in A375 and SK-MEL-28 cells after treatment with increasing doses of NHWD-870 for 24 hours quantified by RT-PCR** (B)** and western blotting** (C)**.** (D)** Cell proliferation of A375 treated with vehicle (DMSO), NHWD-870 (5nM), JQ-1 (500nM) or BMS-986158 (10nM) were quantified by CCK-8 assay. (**E-G**) Scratch-wound healing assay** (E-F)** and Transwell assays **(G)** of A375 cells treated with vehicle, NHWD-870 (5nM), JQ-1 (500nM) or BMS-986158 (10nM). **(H)** Pictures of resected subcutaneous xenografted tumor in nude mice. A375 cells (10^6^) were injected subcutaneously. When the tumor reached 100mm^3^, NHWD-870 (1mg/kg) or vehicle (0.5% methyl cellulose + 0.1% Tween 80) were given orally once a day for five successive days and then two days off. Tumors were resected photographed at day 21 (N = 6 in each group). **(I)** Tumor volume were recorded twice per week by Vernier caliper measurement and calculated as ([length×width^2^]/2).** (J)** Identification by RT-PCR of SPP1 expression in tumor tissue with or without NHWD-870 treatment. All data were presented as mean ± SD of three independent experiments. *, *P* < 0.05; **, *P* < 0.01; ***, *P <* 0.001.

To identify drugs that suppress SPP1 in melanoma, we performed a pilot screening using vemurafenib (BRAF inhibitor), trametinib (MEK inhibitor), and NHWD-870 (BET inhibitor) and found that NHWD-870 inhibited SPP1 expression **(Figure 5A)**. Next, we treated A375 and SK-MEL-28 melanoma cells with different doses of NHWD-870 and obtained consistent results that SPP1 expression was inhibited in a dose-dependent manner **(Figure 5B-C)**. Moreover, widely-used BET inhibitors such as JQ-1 and BMS-986158 30, also decreased SPP1 expression in A375 cells in a dose-dependent manner **(Figure S4)**, suggesting that SPP1 is a common target of BET inhibitors. We previously reported that NHWD-870 indirectly suppressed melanoma growth *in vivo* by inhibiting tumor-associated macrophage proliferation 6. However, the direct effect of NHWD-870 in melanoma growth was not defined. Consistent with our previous findings, BET inhibitors could suppress melanoma proliferation *in vitro* **(Figure 5D)**. BET inhibitors have been reported to have different roles in tumor migration and invasion in different cancers types 31-34. To explore the role of BET inhibitors in melanoma migration and invasion, we performed wound healing and Transwell assays, and results showed delayed wound repair and a smaller number of invasive cells after BET inhibitors treatment **(Figure 5E-G)**.

**Figure 6:**
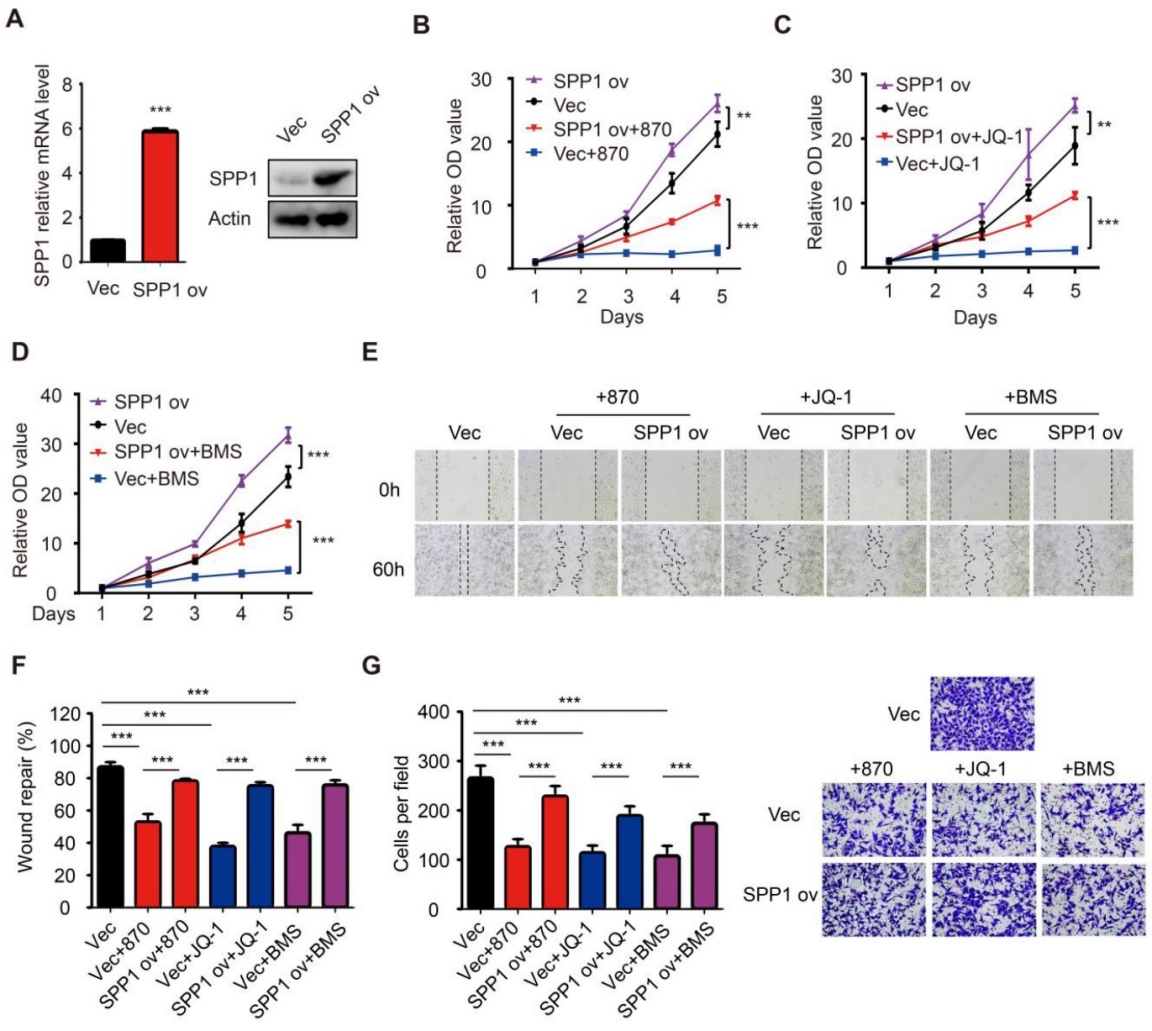
** Overexpression of SPP1 partially reversed the inhibition effects of BET inhibitors in melanoma. (A)** The efficiency of SPP1 overexpression in A375 cells were evaluated by RT-PCR and western blotting.** (B-D)** The influence of SPP1 overexpression on proliferation of A375 cells treated with NHWD-870 (5nM) **(B)**, JQ-1 (500nM) **(C)**, and BMS-986158 (10nM)** (D)**.** (E-G)** The influence of SPP1 overexpression on migration **(E-F)** and invasiveness **(G)** ability of A375 cells treated with NHWD-870 (5nM), JQ-1 (500nM), and BMS-986158 (10nM). All data were presented as mean ± SD of three independent experiments. *, *P* < 0.05; **, *P* < 0.01; ***, *P <* 0.001.

As seen in the *in vitro* experiments, the treatment of mice bearing A375 melanoma cells with NHWD-870 significantly suppressed tumor growth **(Figure 5H-I)**. Besides, NHWD-870 treatment led to a significant decrease in SPP1 expression in these tumors **(Figure 5J)**. To investigate whether SPP1 mediates the effect of BET inhibitors in melanoma, we stably overexpressed SPP1 in A375 cells **(Figure 6A)**. Consistent with previous results, melanoma growth was inhibited by the BET inhibitor and promoted by SPP1 overexpression. Notably, the inhibition could be partially reversed by SPP1 overexpression** (Figure 6B-D)**. Moreover, we found that SPP1 overexpression also rescued the delayed wound repair and reduced invasive ability after treatment with BET inhibitors **(Figure 6E-G)**. These findings collectively suggested that BET inhibitors impede melanoma cell proliferation, migration and invasion by suppressing SPP1 expression.

### BET inhibitor targeted BRD4 indirectly regulates SPP1 expression

**Figure 7:**
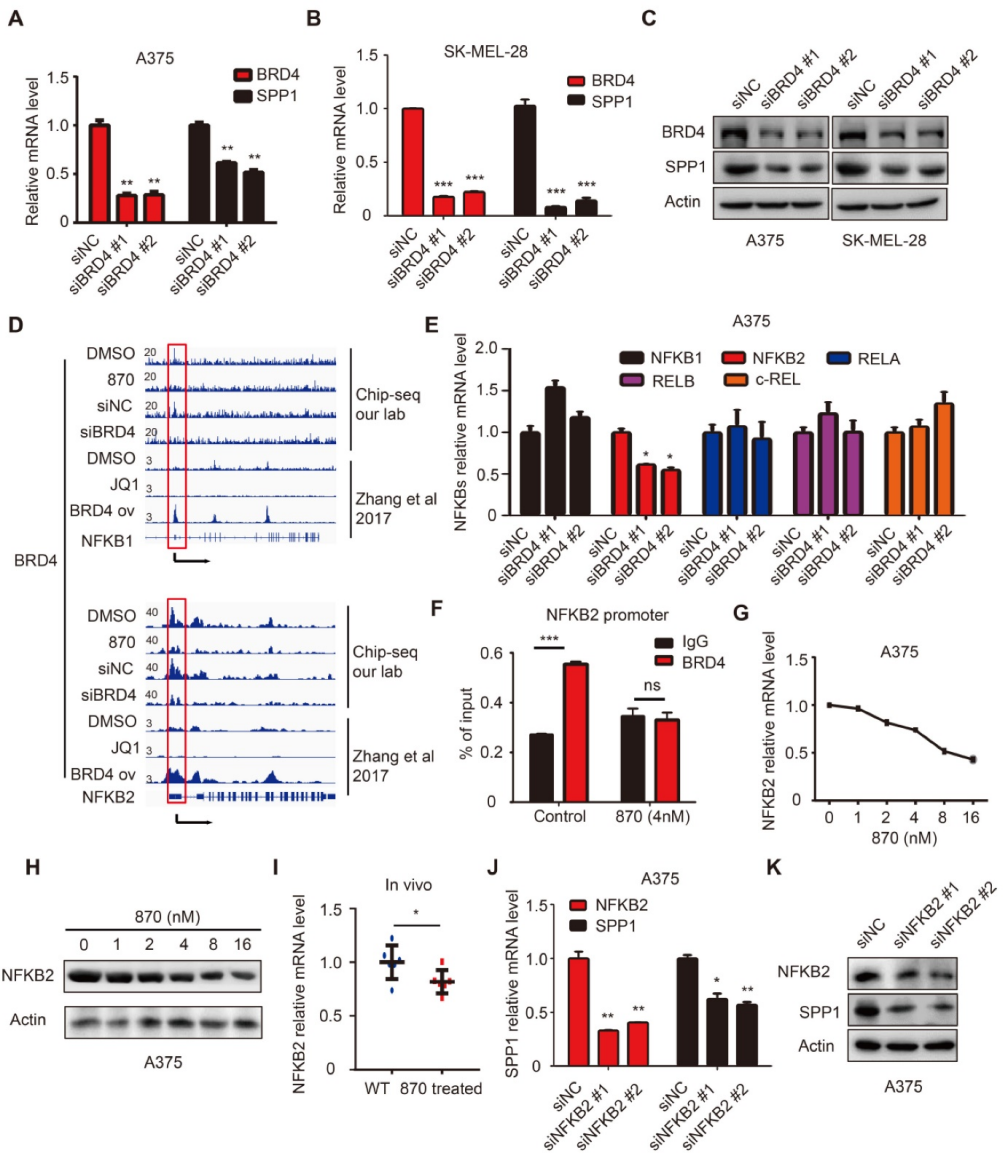
** BET inhibitor inhibits SPP1 expression *via* transcriptional inactivation of NFKB2. (A-C)** BRD4 and SPP1 expression were quantified by RT-PCR** (A-B)** and western blotting** (C)** in A375 and SK-MEL-28 cells 48 hours post-transfection with siNC or siBRD4s.** (D)** BRD4 binding in NFKB1 and NFKB2 promoters among DMSO treated, BET inhibitor (NHWD-870) treated, siNC treated, and siBRD4 treated A375 cells (our CHIP-seq data), or DMSO treated, JQ-1 treated, and BRD4 overexpressed cells (Zhang *et al.*, 2017).** (E)** RT-PCR analysis of NFKB1, NFKB2, RELA, RELB, and c-REL expression in A375 cells 48 hours post-transfection with siNC or siBRD4s.** (F)** Validation of BRD4 binding to the promoter of NFKB2 in A375 and NHWD-870-treated (4nM) A375 cells by ChIP-qPCR. **(G-H)** NFKB2 expression in A375 cells after treatment with increasing dose of NHWD-870 for 24 hours quantified by RT-PCR **(G)** and western blotting **(H)**.** (I)** Identification by RT-PCR of NFKB2 expression in tumor tissue with or without NHWD-870 treatment.** (J-K)** SPP1 expression in A375 cells 48 hours post-transfection with siNC and siNFKB2s quantified by RT-PCR **(J)** and western blotting** (K)**. All data were represented as mean ± SD of three independent experiments. ns, no significance; *, *P* < 0.05; **, *P* < 0.01; ***, *P <* 0.001.

BET inhibitors display a high affinity for bromodomains to competitively suppress BET protein function 35. BET protein consists of BRDT, BRD2, BRD3, and BRD4, among which BRD4 is mainly involved in melanoma progression 7. To determine the role of BRD4 in SPP1 regulation, it was knocked down in SK-MEL-28 and A375 cells, resulting in markedly suppressed SPP1 expression** (Figure 7A-C)**. To investigate the regulation of SPP1 expression by BRD4, we analyzed an existing BRD4 ChIP-seq dataset from Zhang *et al.*'s study and noticed that there was no detectable BRD4-binding on the SPP1 promoter even in BRD4-overexpressing cells **(Figure S5)** 36, as was also shown by our ChIP-seq data** (Figure S5)**. These findings indicated that BRD4 inhibition-induced down-regulation of SPP1 mRNA might occur through an indirect mechanism. To investigate which transcriptional factor might mediate BRD4 inhibition-induced down-regulation of SPP1 mRNA, we searched all potential transcription factors that had been reported to bind with the SPP1 promoter and up-regulate its expression **(Table S9)** 37-49. Our analysis suggested that the NF-κB family played an essential role in SPP1 regulation.

### BET inhibitors suppress SPP1 expression *via* transcriptional inactivation of NFKB2

**Figure 8:**
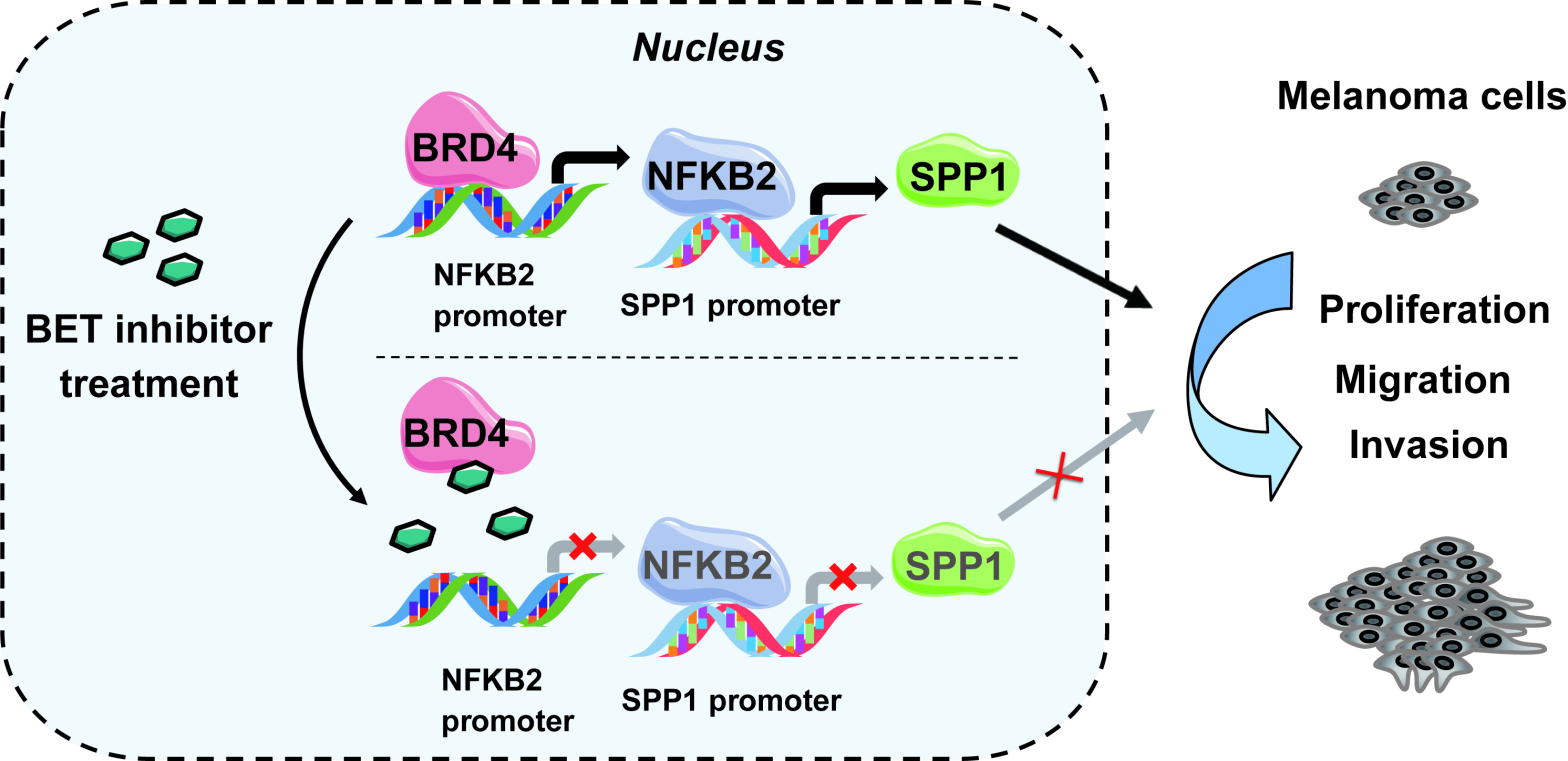
** A model depicting that inhibition of BRD4 represses SPP1 expression by transcriptionally down-regulating NFKB2 in melanoma.** BRD4 promotes NFKB2 expression *via* direct binding to the NFKB2 promoter. Inhibition of BRD4 by BET inhibitor decreases NFKB2 expression, then represses the expression of SPP1, resulting in impeding melanoma proliferation, migration, and invasion.

NF-κB family consists of five inducible transcription factors -- NFKB1, NFKB2, RELA, RELB, and c-REL. To determine which transcription factor of the NF-κB family regulated SPP1 expression, we analyzed our BRD4 ChIP-seq data and found a striking BRD4-binding peak on the NFKB2 gene promoter and a weaker peak on the NFKB1 promoter **(Figure 7D)**. Notably, the binding peak was diminished with NHWD-870 treatment or BRD4 knockdown. These data were consistent with the Zhang laboratory's ChIP-seq data, showing that BRD4 binding on NFKB1 and NFKB2 promoters was diminished by JQ1 treatment and enhanced in BRD4-overexpressing cells **(Figure 7D)** 36. Next, we evaluated the expression of all five NF-κB family genes (NFKB1, NFKB2, RELA, RELB, and c-REL) in BRD4 knockdown A375 cells and found that only NFKB2 was decreased** (Figure 7E)**. ChIP-qPCR was performed to validate the ChIP-Seq data in A375 cells, showing a significant decrease in NFKB2 promoter occupancy by BRD4 after NHWD-870 treatment, compared to control cells **(Figure 7F)**. Also, NHWD-870 treatment led to a considerable loss of NFKB2 in a dose-dependent manner **(Figure 7G-H)**, consistent with JQ-1 or BMS-986158 treatment **(Figure S6A-B)**. The *in vivo* data also demonstrated that NHWD-870 could decrease NFKB2 expression **(Figure 7I)**. Furthermore, silencing NFKB1 or RELA did not affect SPP1 expression** (Figure S7A-D)**, while knockdown of NFKB2 in A375 cells using siRNAs without targeting NFKB1 or RELA **(Figure S8A)** resulted in decreased SPP1 expression **(Figure 6J-K)**. Similar results were observed in SK-MEL-28 melanoma cells **(Figure S8B-D)**. NFKB2 is a member of the noncanonical NF-κB signaling pathway and its role in melanoma was not clarified previously. We found that small interfering RNA-mediated down-regulation of NFKB2 impaired the proliferation, migration and invasion abilities, which mimicked the phenotype of BET inhibitor treatment and SPP1 silencing in melanoma **(Figure S9A-D)**. Overall, these findings have identified a novel mechanism by which BET inhibitors suppress melanoma progression *via* the BRD4-NFKB2-SPP1 axis **(Figure 8)**.

## Discussion

Gene expression profiling and bioinformatics analysis are useful tools for discovering genetic alterations in oncogenesis and identifying prognostic biomarkers. To discover putative tumor oncogenes, Riker *et al.* compared 40 metastatic melanoma samples with 42 primary cutaneous cancers (including 16 melanomas, 11 squamous cell, and 15 basal cell skin cancers) 50. Chen *et al.* identified DEGs between 7 normal skin and 45 primary melanoma samples 51. Our study reanalyzed the GSE15605 and GSE46517 datasets, which included normal skin, primary melanoma and metastatic melanoma data and identified 70 DEGs as potential melanoma driver genes, among which increased SPP1 expression was detected in normal skin < primary melanoma < metastatic melanoma.

SPP1 is a secreted glyco-phosphoprotein and implicated in many biological functions, including cell adhesion, migration, and invasion 17, 22, 27. In our meta-analysis, no difference was observed in IHC staining of SPP1 between primary and metastatic melanoma, but serum SPP1 was higher in metastatic than in primary melanoma patients based on ELISA analysis. No difference was found between healthy individuals and primary melanoma patients by ELISA analysis. However, IHC showed more positive staining in primary melanoma than in nevi, implying that IHC could be used for monitoring SPP1 expression to differentiate nevi from melanoma while ELISA could be more appropriate to distinguish primary from metastatic melanoma.

Next, we analyzed SPP1 expression in different cell lines and found that it was highly expressed in melanoma cell lines compared to HEK-293T and HaCaT cells. These findings were further validated by our analysis of the Xiangya melanoma cohort and HPA database. Previous studies demonstrated that SPP1 could act as a prognostic marker for melanoma 52, 53. Thus, we examined the Xiangya melanoma cohort and found that SPP1 overexpression could predict the unfavorable survival of melanoma patients. We further confirmed that SPP1 acted as a tumorigenic gene and promoted melanoma cell proliferation, migration, and invasion.

Considering the role of SPP1 in melanoma, targeting SPP1 could be beneficial in melanoma therapy. Some small-molecule inhibitors, such as simvastatin and andrographolide, have been reported to inhibit SPP1 transcription in ovarian and breast cancers 54, 55. To explore whether the melanoma drugs could down-regulate SPP1 expression, we performed drug screening using vemurafenib, trametinib, or BET inhibitors and observed that the BET inhibitors suppressed SPP1. We previously demonstrated that NHWD-870, a BET inhibitor, could suppress melanoma growth by blocking cancer cell macrophage interaction. Here we found that NHWD-870 directly inhibited melanoma proliferation, migration, and invasion in an SPP1-dependent manner. Previous studies identified MYC as a crucial target of BET inhibitors, but MYC overexpression failed to rescue the suppression by BET inhibitors in melanoma 7. Our study identified SPP1 as a crucial target of BET inhibitors. Also, BET inhibitors and vemurafenib could act synergistically against BRAF-mutant melanoma but the mechanism was unclear 56. In our study, vemurafenib treatment increased SPP1 expression in a dose-dependent manner. Reversal of vemurafenib-induced SPP1 by BET inhibitors could be a potential mechanism for the synergetic effect of combination therapy.

Furthermore, we found that the BET inhibitor suppressed SPP1 expression in a BRD4-dependent manner. The BRD4 ChIP-seq did not show a BRD4 binding peak on the SPP1 promoter. We hypothesized that BRD4-mediated SPP1 regulation occurred through an indirect mechanism. To identify the potential transcriptional factors of SPP1, we performed a literature search and found NF-κB family members as potential transcriptional factors in our ChIP-seq analysis. The NF-κB signaling consists of a canonical pathway, involving NFKB1, RELA, and c-REL, and a noncanonical pathway including NFKB2 and RELB 57. Our study found that BRD4 silencing in A375 melanoma cells did not affect the expression of transcriptional factors in the canonical NF-κB pathway but affected NFKB2 expression in the noncanonical NF-κB pathway. Activation of NF-κB has been identified to be critical in melanoma progression 58. Gallagher *et al.* previously demonstrated that BET inhibitors suppressed the canonical NF-κB pathway through BRD2 not BRD3/4 59. Our results highlighted the role of BET inhibitors in noncanonical NF-κB pathway through BRD4. Moreover, NFKB2 silencing decreased SPP1 expression, while the silencing of NFKB1 or RELA had no effect on SPP1 expression. NFKB2 silencing resembled the phenotype observed by BET inhibitors and SPP1 silencing in melanoma. Thus, the noncanonical NF-κB, and not canonical pathways, was used by BET inhibitors to regulate SPP1 expression.

MMP2 facilitated tumor growth, invasion, angiogenesis, and metastasis 60-63. Many studies have reported that loss of SPP1 inhibited MMP2 expression 64-67. To verify SPP1's role in mediating MMP2 expression in melanoma, we knocked down SPP1 in A375 or SK-MEL-28 melanoma cells and found decreased MMP2 expression **(Figure S10A-B)**. Further, BET inhibitor treatment, BRD4 silencing or NFKB2 silencing also inhibited MMP2 expression **(Figure S10A-B)**. Overexpression of SPP1 partially reversed MMP2 expression in melanoma cells after BET inhibitor treatment** (Figure S10C)**. These results highlighted the importance of BRD4/NFKB2/SPP1 signaling in melanoma progression.

## Conclusion

In summary, SPP1 expression was positively correlated with melanoma progression, and overexpression of SPP1 predicted poor prognosis in melanoma patients. BET inhibitors suppressed SPP1 expression in a dose-dependent manner, and the inhibition could be partially rescued by SPP1 overexpression. Mechanistically, BET inhibitors suppressed melanoma progression *via* the BRD4/NFKB2/SPP1 axis. Thus, our study highlights SPP1 as an essential target of BET inhibitors and has identified a novel mechanism by which BET inhibitors suppress melanoma progression *via* the noncanonical NF-κB/SPP1 pathway.

## Supplementary Material

Supplementary figures and tables.

## Abbreviations

SPP1: secreted phosphoprotein 1
BRAF: B-Raf proto-oncogene
MEK: MAP kinase-ERK kinase
EZH2: enhancer of zeste 2 polycomb repressive complex 2 subunit
BET: bromodomain and extra-terminal domain
BRD4: bromodomain containing 4
CKD: cyclin-dependent kinase
NF-κB: nuclear factor kappa B
GEO: Gene Expression Omnibus
TCGA: The Cancer Genome Atlas
DEG: differentially expressed gene
IHC: immunohistochemistry
ELISA: enzyme-linked immunosorbent assay
PVDF: polyvinylidene fluoride
RNA-seq: RNA-sequencing
ChIP: Chromatin immunoprecipitation

## References

[1] Schadendorf D, van Akkooi ACJ, Berking C, Griewank KG, Gutzmer R, Hauschild A (2018). Melanoma. Lancet.

[2] El-Deiry WS, Goldberg RM, Lenz HJ, Shields AF, Gibney GT, Tan AR (2019). The current state of molecular testing in the treatment of patients with solid tumors, 2019. CA Cancer J Clin.

[3] Fontanals-Cirera B, Hasson D, Vardabasso C, Di Micco R, Agrawal P, Chowdhury A (2017). Harnessing BET Inhibitor Sensitivity Reveals AMIGO2 as a Melanoma Survival Gene. Mol Cell.

[4] Cochran AG, Conery AR, Sims RJ 3rd (2019). Bromodomains: a new target class for drug development. Nat Rev Drug Discov.

[5] Zhang Z, Ma P, Jing Y, Yan Y, Cai MC, Zhang M (2016). BET Bromodomain Inhibition as a Therapeutic Strategy in Ovarian Cancer by Downregulating FoxM1. Theranostics.

[6] Yin M, Guo Y, Hu R, Cai WL, Li Y, Pei S (2020). Potent BRD4 inhibitor suppresses cancer cell-macrophage interaction. Nat Commun.

[7] Segura MF, Fontanals-Cirera B, Gaziel-Sovran A, Guijarro MV, Hanniford D, Zhang G (2013). BRD4 sustains melanoma proliferation and represents a new target for epigenetic therapy. Cancer Res.

[8] Cheng W, Ren X, Zhang C, Cai J, Liu Y, Han S (2016). Bioinformatic profiling identifies an immune-related risk signature for glioblastoma. Neurology.

[9] Wei CY, Zhu MX, Lu NH, Peng R, Yang X, Zhang PF (2019). Bioinformatics-based analysis reveals elevated MFSD12 as a key promoter of cell proliferation and a potential therapeutic target in melanoma. Oncogene.

[10] Tu Y, Chen C, Fan G (2019). Association between the expression of secreted phosphoprotein - related genes and prognosis of human cancer. BMC Cancer.

[11] Huang RH, Quan YJ, Chen JH, Wang TF, Xu M, Ye M (2017). Osteopontin Promotes Cell Migration and Invasion, and Inhibits Apoptosis and Autophagy in Colorectal Cancer by activating the p38 MAPK Signaling Pathway. Cell Physiol Biochem.

[12] Zeng B, Zhou M, Wu H, Xiong Z (2018). SPP1 promotes ovarian cancer progression via Integrin beta1/FAK/AKT signaling pathway. Onco Targets Ther.

[13] Chiou J, Chang YC, Tsai HF, Lin YF, Huang MS, Yang CJ (2019). Follistatin-like Protein 1 Inhibits Lung Cancer Metastasis by Preventing Proteolytic Activation of Osteopontin. Cancer Res.

[14] Rhodes DR, Yu J, Shanker K, Deshpande N, Varambally R, Ghosh D (2004). ONCOMINE: a cancer microarray database and integrated data-mining platform. Neoplasia.

[15] Tang Z, Kang B, Li C, Chen T, Zhang Z (2019). GEPIA2: an enhanced web server for large-scale expression profiling and interactive analysis. Nucleic Acids Res.

[16] Barak V, Frenkel S, Kalickman I, Maniotis AJ, Folberg R, Pe'er J (2007). Serum markers to detect metastatic uveal melanoma. Anticancer Res.

[17] Elias EG, Hasskamp JH, Sharma BK (2010). Cytokines and growth factors expressed by human cutaneous melanoma. Cancers (Basel).

[18] Haritoglou I, Wolf A, Maier T, Haritoglou C, Hein R, Schaller UC (2009). Osteopontin and 'melanoma inhibitory activity': comparison of two serological tumor markers in metastatic uveal melanoma patients. Ophthalmologica.

[19] Kadkol SS, Lin AY, Barak V, Kalickman I, Leach L, Valyi-Nagy K (2006). Osteopontin expression and serum levels in metastatic uveal melanoma: a pilot study. Invest Ophthalmol Vis Sci.

[20] Kiss T, Ecsedi S, Vizkeleti L, Koroknai V, Emri G, Kovacs N (2015). The role of osteopontin expression in melanoma progression. Tumour Biol.

[21] Maier T, Laubender RP, Sturm RA, Klingenstein A, Korting HC, Ruzicka T (2012). Osteopontin expression in plasma of melanoma patients and in melanocytic tumours. J Eur Acad Dermatol Venereol.

[22] Packer L, Pavey S, Parker A, Stark M, Johansson P, Clarke B (2006). Osteopontin is a downstream effector of the PI3-kinase pathway in melanomas that is inversely correlated with functional PTEN. Carcinogenesis.

[23] Reiniger IW, Wolf A, Welge-Lussen U, Mueller AJ, Kampik A, Schaller UC (2007). Osteopontin as a serologic marker for metastatic uveal melanoma: results of a pilot study. Am J Ophthalmol.

[24] Soikkeli J, Lukk M, Nummela P, Virolainen S, Jahkola T, Katainen R (2007). Systematic search for the best gene expression markers for melanoma micrometastasis detection. J Pathol.

[25] Song J, Merbs SL, Sokoll LJ, Chan DW, Zhang Z (2019). A multiplex immunoassay of serum biomarkers for the detection of uveal melanoma. Clin Proteomics.

[26] Xu Zhang XX, Bin Li, Fei Gao, Zhibao Zhang, Liang Li (2012). Expression of osteopontin in different metastatic potential uveal melanoma and its significance. Chin J Exp Ophthalmol.

[27] Zhou Y, Dai DL, Martinka M, Su M, Zhang Y, Campos EI (2005). Osteopontin expression correlates with melanoma invasion. J Invest Dermatol.

[28] Filia A, Elliott F, Wind T, Field S, Davies J, Kukalizch K (2013). Plasma osteopontin concentrations in patients with cutaneous melanoma. Oncol Rep.

[29] Rangel J, Nosrati M, Torabian S, Shaikh L, Leong SP, Haqq C (2008). Osteopontin as a molecular prognostic marker for melanoma. Cancer.

[30] Helin K, Dhanak D (2013). Chromatin proteins and modifications as drug targets. Nature.

[31] Bid HK, Phelps DA, Xaio L, Guttridge DC, Lin J, London C (2016). The Bromodomain BET Inhibitor JQ1 Suppresses Tumor Angiogenesis in Models of Childhood Sarcoma. Mol Cancer Ther.

[32] Wang J, Yu Q, Qiu Z, Dai T, Wang S, Yang X (2020). The combined effect of epigenetic inhibitors for LSD1 and BRD4 alters prostate cancer growth and invasion. Aging (Albany NY).

[33] Wang L, Xu M, Kao CY, Tsai SY, Tsai MJ (2020). Small molecule JQ1 promotes prostate cancer invasion via BET-independent inactivation of FOXA1. J Clin Invest.

[34] Zhou S, Zhang S, Wang L, Huang S, Yuan Y, Yang J (2020). BET protein inhibitor JQ1 downregulates chromatin accessibility and suppresses metastasis of gastric cancer via inactivating RUNX2/NID1 signaling. Oncogenesis.

[35] Shi J, Vakoc CR (2014). The mechanisms behind the therapeutic activity of BET bromodomain inhibition. Mol Cell.

[36] Zhang P, Wang D, Zhao Y, Ren S, Gao K, Ye Z (2017). Intrinsic BET inhibitor resistance in SPOP-mutated prostate cancer is mediated by BET protein stabilization and AKT-mTORC1 activation. Nat Med.

[37] Bidder M, Shao JS, Charlton-Kachigian N, Loewy AP, Semenkovich CF, Towler DA (2002). Osteopontin transcription in aortic vascular smooth muscle cells is controlled by glucose-regulated upstream stimulatory factor and activator protein-1 activities. J Biol Chem.

[38] Cheng HC, Liu YP, Shan YS, Huang CY, Lin FC, Lin LC (2013). Loss of RUNX3 increases osteopontin expression and promotes cell migration in gastric cancer. Carcinogenesis.

[39] Lee JM, Libermann TA, Cho JY (2010). The synergistic regulatory effect of Runx2 and MEF transcription factors on osteoblast differentiation markers. J Periodontal Implant Sci.

[40] Lyle AN, Remus EW, Fan AE, Lassegue B, Walter GA, Kiyosue A (2014). Hydrogen peroxide regulates osteopontin expression through activation of transcriptional and translational pathways. J Biol Chem.

[41] Renault MA, Jalvy S, Potier M, Belloc I, Genot E, Dekker LV (2005). UTP induces osteopontin expression through a coordinate action of NFkappaB, activator protein-1, and upstream stimulatory factor in arterial smooth muscle cells. J Biol Chem.

[42] Samant RS, Clark DW, Fillmore RA, Cicek M, Metge BJ, Chandramouli KH (2007). Breast cancer metastasis suppressor 1 (BRMS1) inhibits osteopontin transcription by abrogating NF-kappaB activation. Mol Cancer.

[43] Sharma P, Kumar S, Kundu GC (2010). Transcriptional regulation of human osteopontin promoter by histone deacetylase inhibitor, trichostatin A in cervical cancer cells. Mol Cancer.

[44] Sowa AK, Kaiser FJ, Eckhold J, Kessler T, Aherrahrou R, Wrobel S (2013). Functional interaction of osteogenic transcription factors Runx2 and Vdr in transcriptional regulation of Opn during soft tissue calcification. Am J Pathol.

[45] Takami Y, Russell MB, Gao C, Mi Z, Guo H, Mantyh CR (2007). Sp1 regulates osteopontin expression in SW480 human colon adenocarcinoma cells. Surgery.

[46] Wang YG, Qu XH, Yang Y, Han XG, Wang L, Qiao H (2016). AMPK promotes osteogenesis and inhibits adipogenesis through AMPK-Gfi1-OPN axis. Cell Signal.

[47] Zhang J, Yamada O, Kida S, Matsushita Y, Hattori T (2016). Down-regulation of osteopontin mediates a novel mechanism underlying the cytostatic activity of TGF-beta. Cell Oncol (Dordr).

[48] Zhang Q, Wang C, Tang Y, Zhu Q, Li Y, Chen H (2019). High glucose up-regulates osteopontin expression by FoxO1 activation in macrophage. J Endocrinol.

[49] Zhao W, Wang L, Zhang M, Wang P, Zhang L, Yuan C (2011). NF-kappaB- and AP-1-mediated DNA looping regulates osteopontin transcription in endotoxin-stimulated murine macrophages. J Immunol.

[50] Della Volpe N, Bianco L, Bonuso C, Annecchiarico M, Di Silverio P, Caiazza A (2008). Rare ileal localisation of angiolipoma presenting as chronic haemorrhage and severe anaemia: a case report. J Med Case Rep.

[51] Chen J, Sun W, Mo N, Chen X, Yang L, Tu S (2020). Identification of key genes involved in the pathogenesis of cutaneous melanoma using bioinformatics analysis. J Int Med Res.

[52] Kashani-Sabet M, Nosrati M, Miller JR 3rd, Sagebiel RW, Leong SPL, Lesniak A (2017). Prospective Validation of Molecular Prognostic Markers in Cutaneous Melanoma: A Correlative Analysis of E1690. Clin Cancer Res.

[53] Kashani-Sabet M, Venna S, Nosrati M, Rangel J, Sucker A, Egberts F (2009). A multimarker prognostic assay for primary cutaneous melanoma. Clin Cancer Res.

[54] Kumar S, Patil HS, Sharma P, Kumar D, Dasari S, Puranik VG (2012). Andrographolide inhibits osteopontin expression and breast tumor growth through down regulation of PI3 kinase/Akt signaling pathway. Curr Mol Med.

[55] Matsuura M, Suzuki T, Saito T (2010). Osteopontin is a new target molecule for ovarian clear cell carcinoma therapy. Cancer Sci.

[56] Paoluzzi L, Hanniford D, Sokolova E, Osman I, Darvishian F, Wang J (2016). BET and BRAF inhibitors act synergistically against BRAF-mutant melanoma. Cancer Med.

[57] Sun SC (2017). The non-canonical NF-kappaB pathway in immunity and inflammation. Nat Rev Immunol.

[58] Zhou J, Jin B, Jin Y, Liu Y, Pan J (2017). The antihelminthic drug niclosamide effectively inhibits the malignant phenotypes of uveal melanoma *in vitro* and *in vivo*. Theranostics.

[59] Gallagher SJ, Mijatov B, Gunatilake D, Gowrishankar K, Tiffen J, James W (2014). Control of NF-kB activity in human melanoma by bromodomain and extra-terminal protein inhibitor I-BET151. Pigment Cell Melanoma Res.

[60] Detry B, Erpicum C, Paupert J, Blacher S, Maillard C, Bruyere F (2012). Matrix metalloproteinase-2 governs lymphatic vessel formation as an interstitial collagenase. Blood.

[61] Giannelli G, Falk-Marzillier J, Schiraldi O, Stetler-Stevenson WG, Quaranta V (1997). Induction of cell migration by matrix metalloprotease-2 cleavage of laminin-5. Science.

[62] Pereira MS, Celeiro SP, Costa AM, Pinto F, Popov S, de Almeida GC (2019). Loss of SPINT2 expression frequently occurs in glioma, leading to increased growth and invasion via MMP2. Cell Oncol (Dordr).

[63] Wang X, Hu Z, Wang Z, Cui Y, Cui X (2019). Angiopoietin-like protein 2 is an important facilitator of tumor proliferation, metastasis, angiogenesis and glycolysis in osteosarcoma. Am J Transl Res.

[64] Ferreira LB, Tavares C, Pestana A, Pereira CL, Eloy C, Pinto MT (2016). Osteopontin-a splice variant is overexpressed in papillary thyroid carcinoma and modulates invasive behavior. Oncotarget.

[65] Seo KW, Lee SJ, Ye BH, Kim YW, Bae SS, Kim CD (2015). Mechanical stretch enhances the expression and activity of osteopontin and MMP-2 via the Akt1/AP-1 pathways in VSMC. J Mol Cell Cardiol.

[66] Wu XL, Lin KJ, Bai AP, Wang WX, Meng XK, Su XL (2014). Osteopontin knockdown suppresses the growth and angiogenesis of colon cancer cells. World J Gastroenterol.

[67] Xu J, Sun Y, Wang T, Liu G (2013). Prevention of neointimal hyperplasia in balloon-injured rat carotid artery via small interference RNA mediated downregulation of osteopontin gene. Mol Cell Biochem.

