# Supplementary material for: BET inhibitor suppresses melanoma progression via the noncanonical NF-κB/SPP1 pathway
Source: Theranostics. 2020 Sep 15;10(25):11428–43. doi: 10.7150/thno.47432 (PMC7546000; doi:10.7150/thno.47432)
Supplement: Supplementary file 1 — Supplementary figures and tables. [file thnov10p11428s1.pdf]

BET inhibitor suppresses melanoma progression via the noncanonical NF-κB/SPP1 pathway

Guangtong Deng et al.

Supplementary figures 1-10
Supplementary tables 1-9

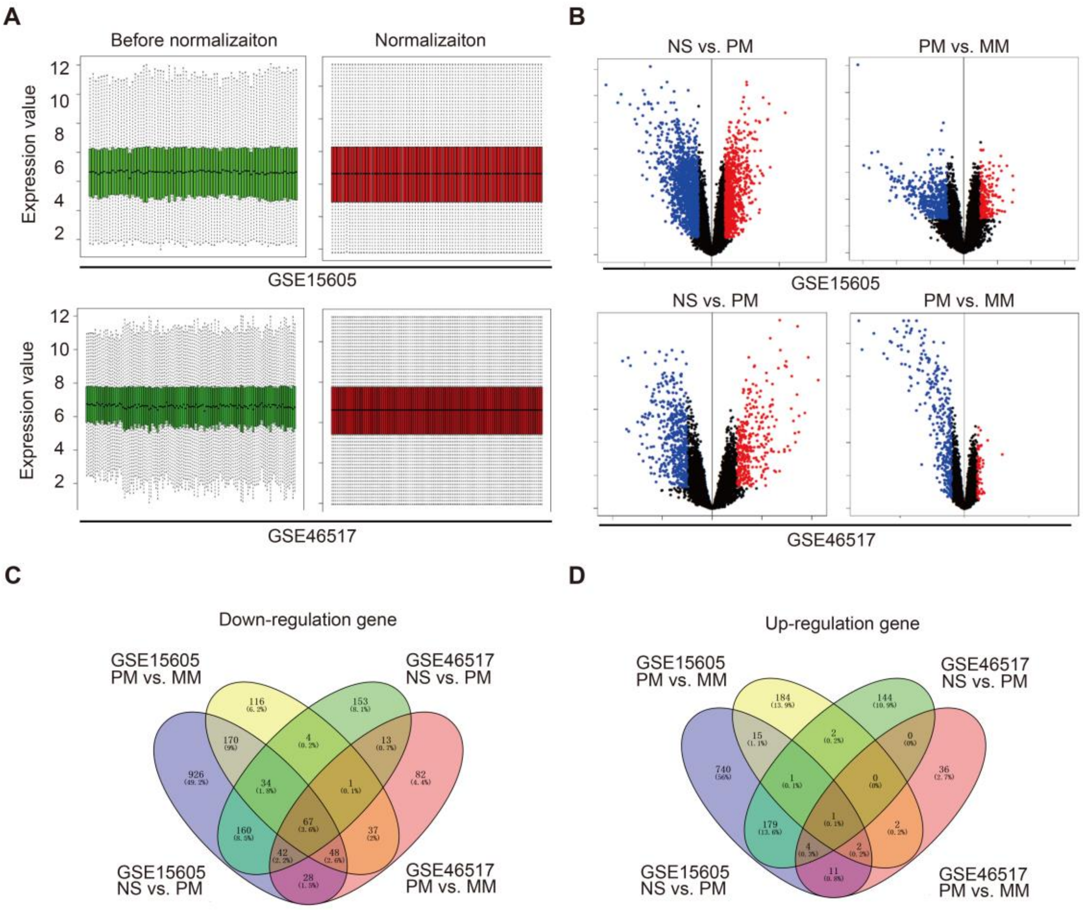

Figure S1. Identification of SPP1 as a potential melanoma driver. (**A**) normalization of GSE15605 and GSE46517. (**B**) volcano plot of GSE15605 and GSE46517 among normal skin, primary, and metastatic melanoma. (**C-D**) Venn diagram of down-regulated genes (**C**) and up-regulated genes (**D**) from normal skin, primary melanoma, and metastatic melanoma in both GSE15605 and GSE46517.

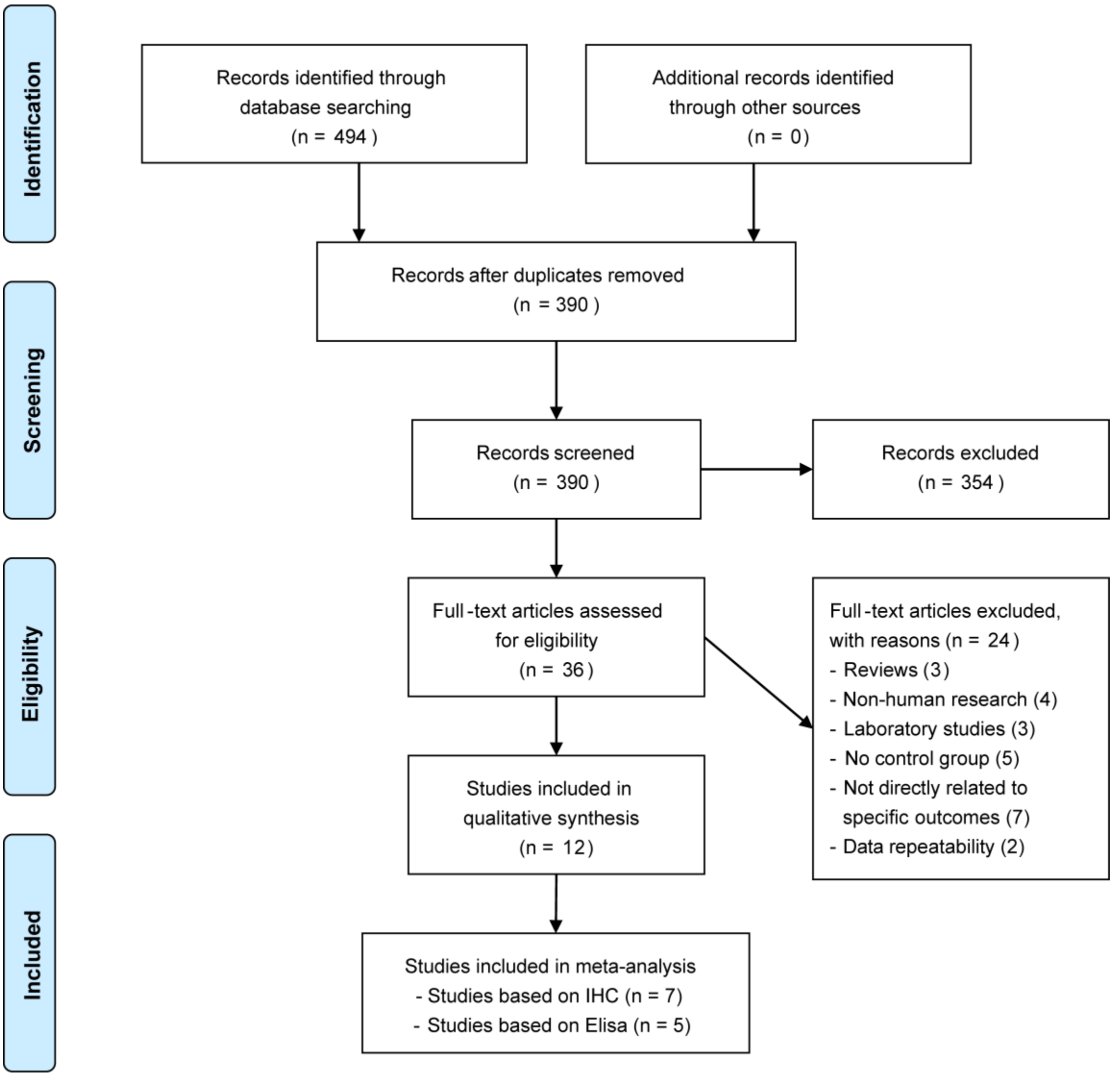

Figure S2. Flowchart of literature search and study selection process.

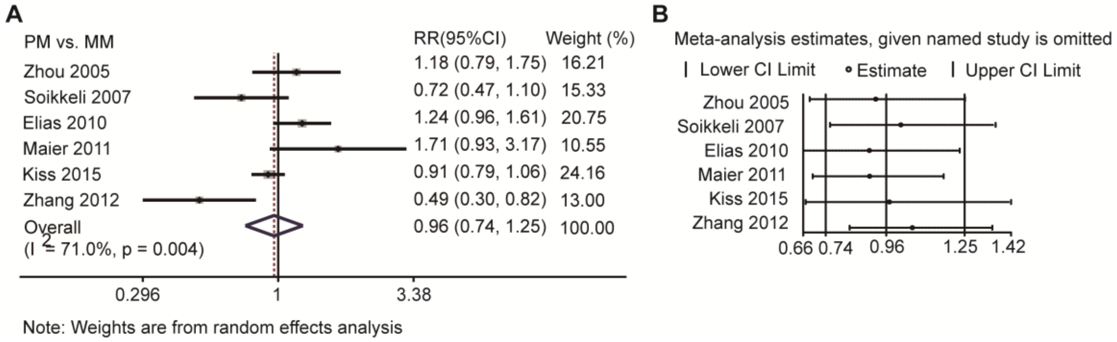

Figure S3. Forest plot (**A**) and sensitivity analysis (**B**) of SPP1 expression between primary and metastatic melanoma based on immunohistochemistry.

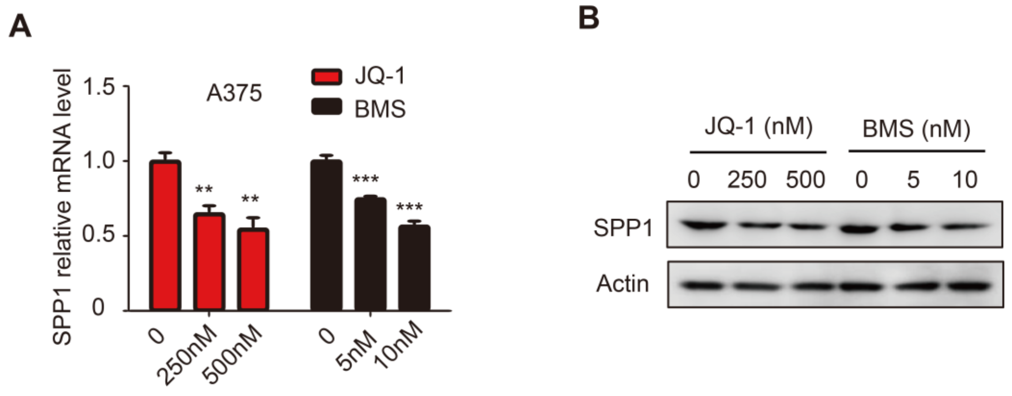

Figure S4. SPP1 expression was decreased after BET inhibitors treatment. SPP1 expression quantified by RT-PCR (**A**) and western blotting (**B**) after treatment with JQ-1 or BMS-986158. All data were represented as mean ± SD of three independent experiments. **, $P < 0.01$; ***, $P < 0.001$.

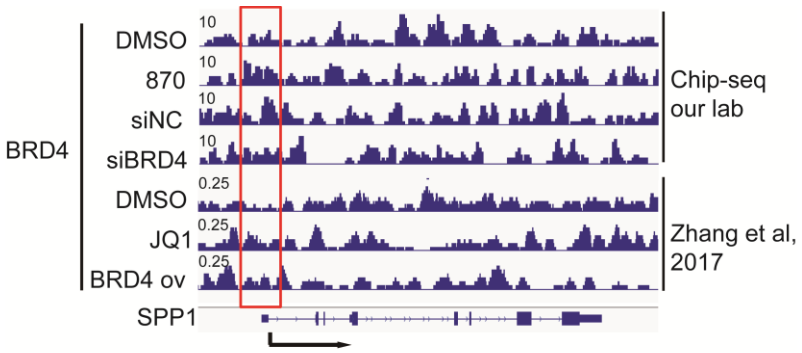

Figure S5. BRD4 regulates SPP1 expression in an indirect manner. BRD4 binding in SPP1 promoter among DMSO treated, BET inhibitor (NHWD-870) treated, siNC treated, and siBRD4 treated A375 cells (our CHIP-seq data, upper). BRD4 binding in SPP1 promoter in DMSO treated, JQ-1 treated, and BRD4 overexpressed cells (Zhang et al., 2017, down).

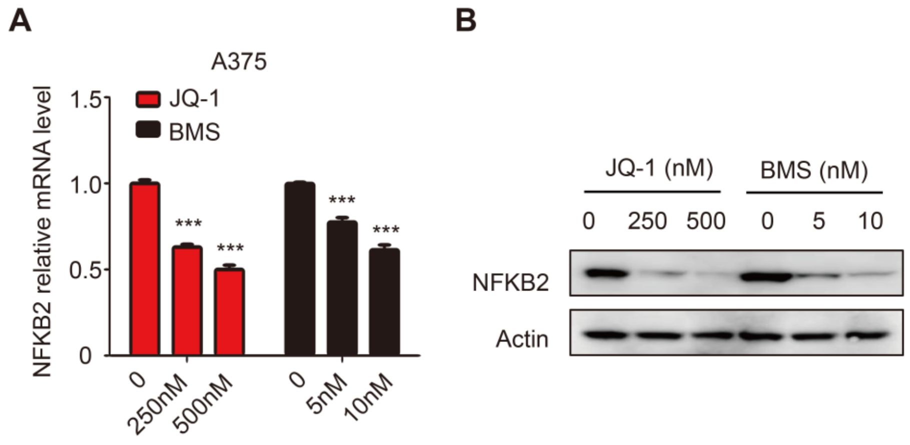

Figure S6. NFKB2 expression was decreased after BET inhibitors treatment. NFKB2 expression in A375 cells quantified by RT-PCR (**A**) or western blotting (**B**) after treatment with JQ-1 or BMS-986158.

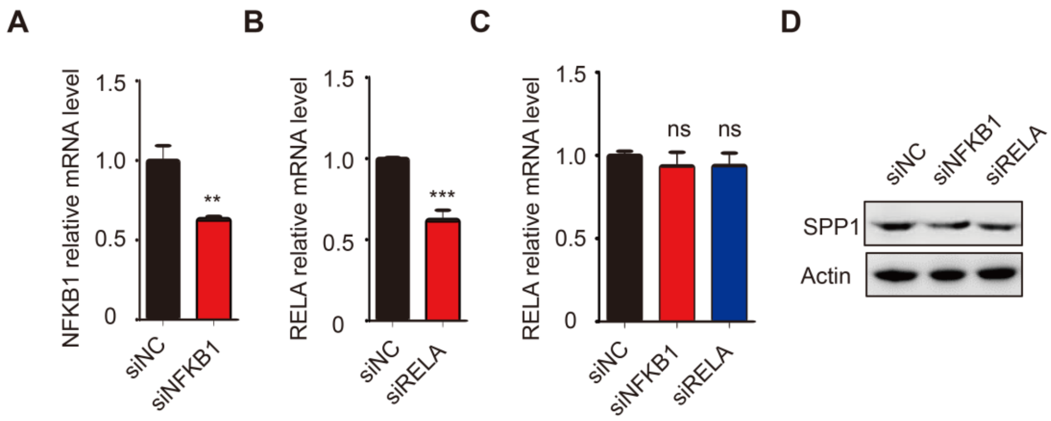

Figure S7. SPP1 expression is not affected by NFKB1 or RELA silencing. (**A-B**) Knockdown efficiency of NFKB1 (**A**) and RELA (**B**) quantified by RT-PCR in A375 cells 48 hours post-transfection with siNC, siNFKB1, or siRELA. (**C-D**) SPP1 expression were quantified by RT-PCR (**C**) and western blotting (**D**) in A375 cells 48 hours post-transfection with siNC, siNFKB1, or siRELA. All data were represented as mean ± SD of three independent experiments. **, P < 0.01; ***, P < 0.001.

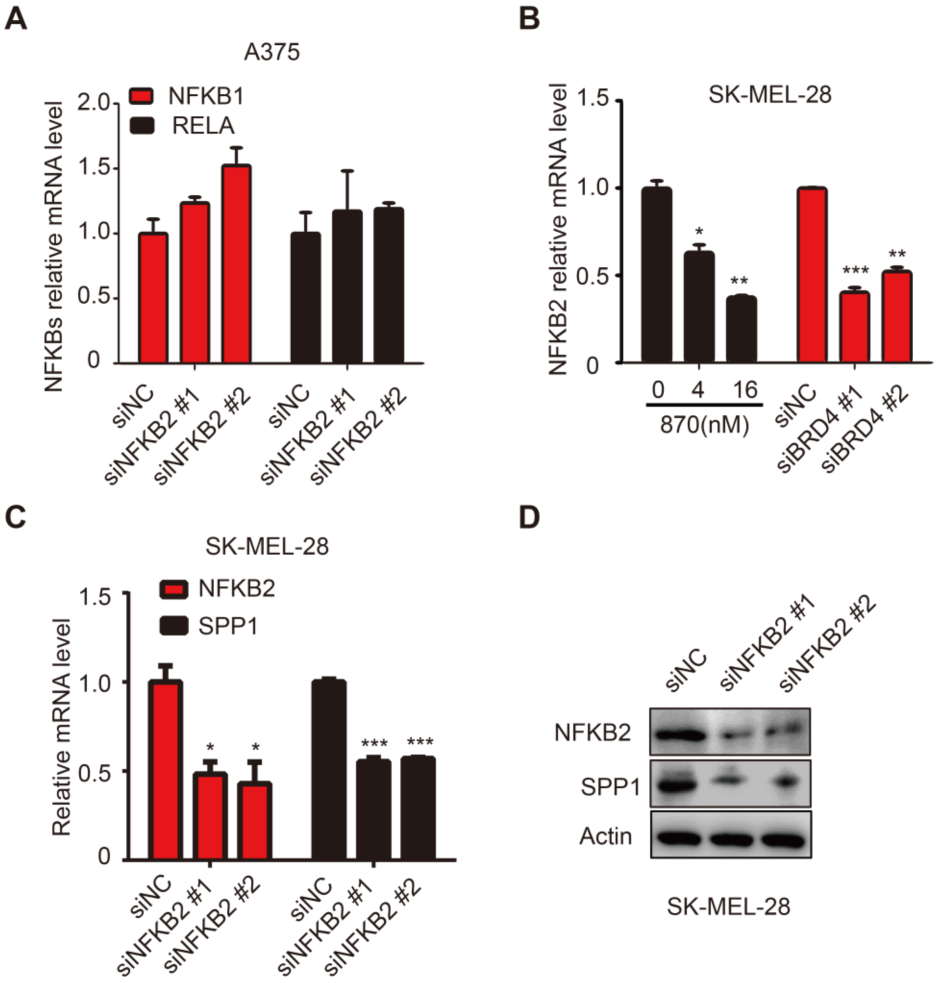

Figure S8. BET inhibitor inhibits SPP1 expression via transcriptional inactivation of NFKB2. (**A**) RT-PCR analysis of NFKB1 and RELA expression in A375 cells 48 hours post-transfection with siNC and siNFKB2s. (**B**) RT-PCR analysis of NFKB2 expression in SK-MEL-28 cells treated with increasing dose of NHWD-870 for 24 hours or 48 hours post-transfection with siNC or siBRD4s. (**C-D**) NFKB2 and SPP1 expression in SK-MEL-28 cells 48 hours post-transfection with siNC and siNFKB2s quantified by RT-PCR (**C**) and western blotting (**D**). All data were represented as mean ± SD of three independent experiments . *, $P < 0.05$; **, $P < 0.01$; ***, $P < 0.001$.

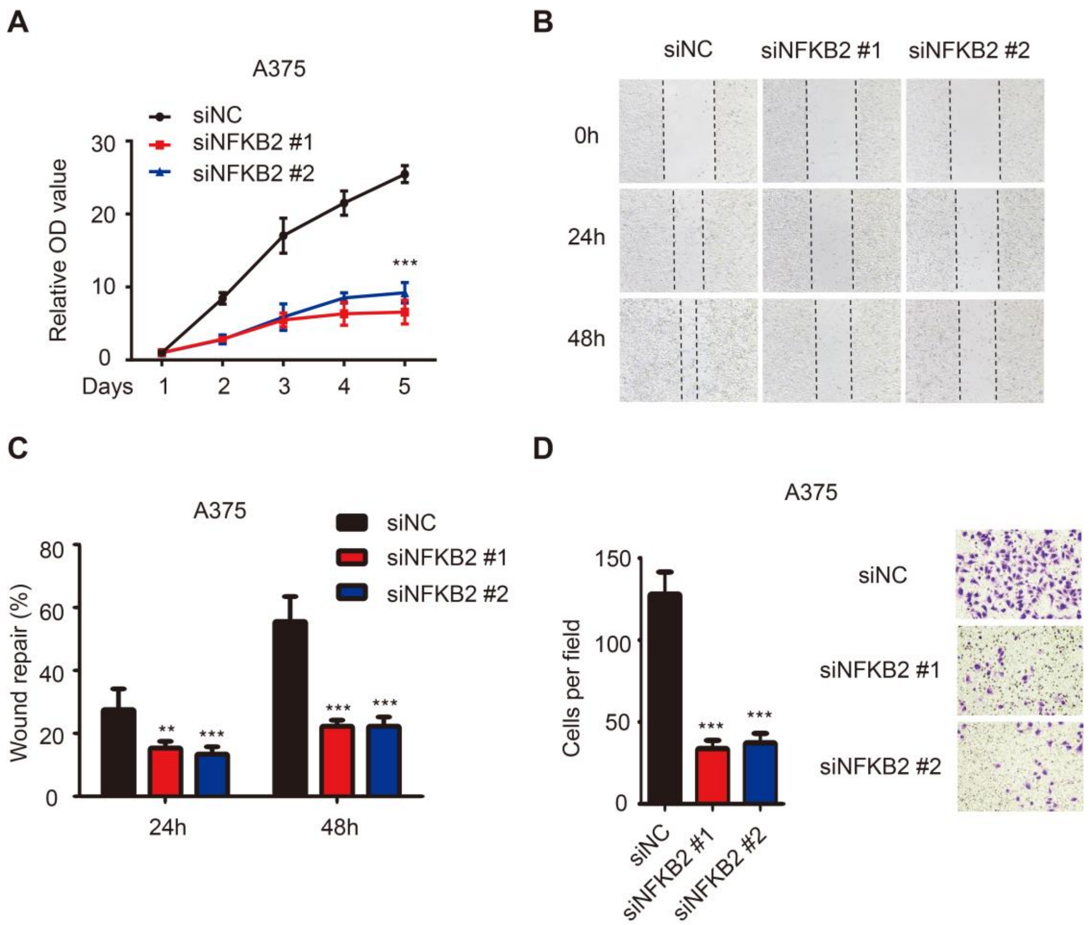

Figure S9. Knockdown of NFKB2 inhibits melanoma cell proliferation, migration and invasion. (**A**) Cell proliferation of A375 cells transfected with siNC, siNFKB2 #1, and siNFKB2 #2 were quantified by CCK-8. (**B-C**) Scratch-wound healing assay of A375 cells transduced with transfected with siNC, siNFKB2 #1, and siNFKB2 #2. (**D**) Invasiveness of A375 cells transfected with siNC, siNFKB2 #1 and siNFKB2 #2 were assessed by Transwell assays. All data were represented as mean ± SD of three independent experiments. **, $P < 0.01$; ***, $P < 0.001$.

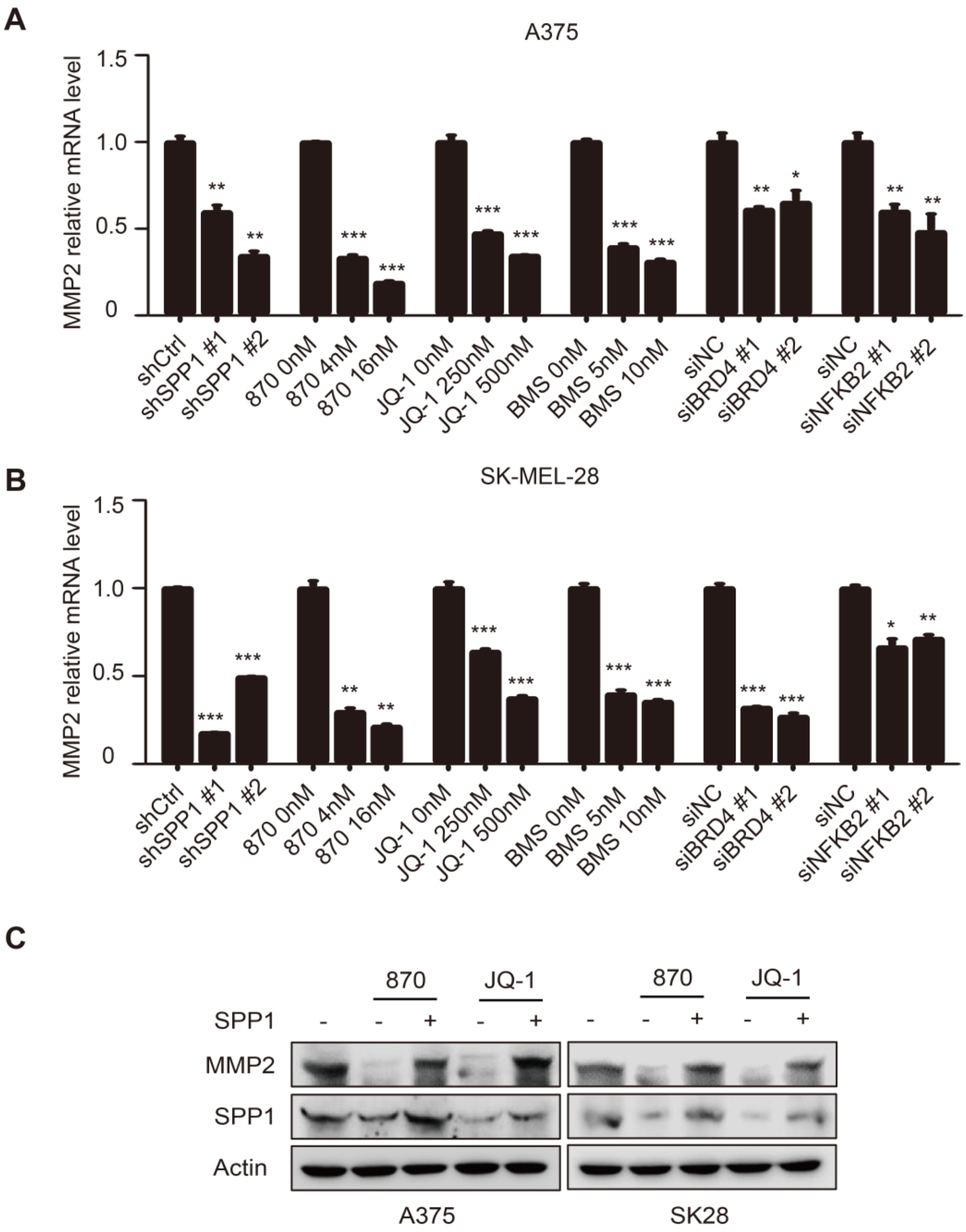

Figure S10. BRD4/ NFKB2/SPP1 signaling regulates the MMP2 activity. (**A-B**) RT-PCR analysis of MMP2 expression in A375 cells (**A**) and SK-MEL-28 cells (**B**) post-transfection with shSPP1, siBRD4, siNFKB2, corresponding control, or treated with increasing dose of BET inhibitors for 24 hours. (**C**) The influence of SPP1 overexpression on MMP2 expression in A375 cells treated with NHWD-870 (5nM) or JQ-1 (500nM). All data were represented as mean ± SD of three independent experiments. *, $P < 0.05$; **, $P < 0.01$; ***, $P < 0.001$.

Table S1. Search strategies

Databases	Records identified	Search strategies
Pubmed	152	#1 "Melanoma"[Mesh]<br>#2 melanoma$[Title/Abstract]<br>#3 #1 OR #2<br>#4 "Skin"[Mesh]<br>#5 ((skin or epiderm$ or derm$ or cutaneous))<br>#6 #4 OR #5<br>#7 #3 AND #6<br>#8 malignant melanoma$<br>#9 "Skin Neoplasms"[Mesh]<br>#10 skin cancer$<br>#11 skin neoplas$<br>#12 #7 OR #8 OR #9 OR #10 OR #11<br>#13 spp1 OR (secreted phosphoprotein 1) OR osteopontin OR bnsp OR bspi OR (eta-1) OR opn<br>#14 #12 AND #13
EMBASE	157	#1 'melanoma'/exp<br>#2 melanoma$<br>#3 #1 OR #2<br>#4 'skin'/exp<br>#5 skin OR epiderm$ OR derm$ OR cutaneous<br>#6 #4 OR #5<br>#7 #3 AND #6<br>#8 'malignant melanoma$'<br>#9 'skin tumor'/exp<br>#10 'skin cancer$<br>#11 'skin neoplas$'<br>#12 #7 OR #8 OR #9 OR #10 OR #11<br>#13 spp1 OR 'secreted phosphoprotein 1' OR osteopontin OR bnsp OR bspi OR 'eta-1' OR opn<br>#14 #12 AND #13
Web of Science	185	#1 **TOPIC:** (melanoma$)<br>#2 **TOPIC:** (skin or epiderm$ or derm$ or cutaneous)<br>#3 #1 AND #2<br>#4 **TOPIC:** (malignant melanoma$)<br>#5 **TOPIC:** ("Skin Neoplasms")<br>#6 **TOPIC:** (skin cancer$)<br>#7 **TOPIC:** (skin neoplas$)<br>#8 #3 OR #4 OR #5 OR #6 OR #7<br>#9 **TOPIC:** (spp1 OR (secreted phosphoprotein 1) OR osteopontin OR bnsp OR bspi OR (eta-1) OR opn)<br>#10 #8 AND #9

Table S2. Interfering sequence of siRNAs.

Gene symbol	Target sequence
siBRD4 #1	GCGUUUCCACGGUACCAAATT
siBRD4 #2	CCGUGAUGCUCAGGAGUUUTT
siNFKB1	GUCACUCUAACGUAUGCAAUU
siNFKB2 #1	CCACAGATGTGCATAAACA
siNFKB2 #2	GGACATGACTGCTCAATTT
siRELA	GCCCUAUCCCUUUACGUCA

Table S3. Sequence of primers used for RT-PCR.

Gene symbol	Forward primer	Reverse primer
SPP1	CGAGGTGATAGTGTGGTTTATGG	GCACCATTCAACTCCTCGCTTTC
BRD4	ACCTCCAACCCTAACAAGCC	TTTCCATAGTGTCTTGAGCACC
NFKB1	GCAGCACTACTTCTTGACCACC	TCTGCTCCTGAGCATTGACGTC
NFKB2	GGCAGACCAGTGTCATTGAGCA	CAGCAGAAAGCTCACCACACTC
RELA	CTGCAGTTTGATGATGAAGA	TAGGCGAGTTATAGCCTCAG
RELB	TGTGGTGAGGATCTGCTTCCAG	TCGGCAAATCCGCAGCTCTGAT
C-REL	AGTTGCGGAGACCTTCTGACCA	CGTGATCCTGGCACAGTTTCTG
MMP2	AGCGAGTGGATGCCGCCTTTAA	CATTCCAGGCATCTGCGATGAG
GAPDH	AATCCCATCACCATCTTCCA	GTCATCATATTTGGCAGGTT

Table S4. Characteristics of enrolled studies.

Included studies	Year	Country	Detect methods	Sample origin	Controls	PM/DF/MM patients	antibody	Quality
Zhou	2005	Canada	IHC	Tissue	15	PM: 68, MM:18	Monoclonal anti-OPN antibody	8
Kadkol	2006	USA	Elisa	Serum	30	DF: 37, MM: 15	Elisa assay	7
Packer	2006	Australia	IHC	Tissue	29	PM: 67, MM: 19	Monoclonal OPN antibody	7
Reiniger	2006	Germany	Elisa	Serum	7	PM:19, MM: 8	Elisa Kits	7
Barak	2007	Israel	Elisa	Serum	53	DF: 38, MM: 18	Elisa Kits	7
Soikkeli	2007	Finland	IHC	Tissue	12	PM: 12, MM: 14	Mouse-anti-monoclonal antibody	7
Haritoglou	2009	Germany	Elisa	Serum	-	PM: 18, MM: 14	Elisa Kits	7
Elias	2010	USA	IHC	Tissue	-	PM: 12, MM: 15	Monoclonal anti-OPN antibody	7
Maier	2011	Germany	IHC	Tissue	24	PM:45, MM: 18	Monoclonal antibody	9
Zhang	2012	China	IHC	Tissue	-	PM: 14, MM: 10	Rabbit polyclonal anti-OPN IgG	9
Kiss	2015	Hungary	IHC	Tissue	-	PM: 56, MM: 37	Rabbit polyclonal anti-OPN IgG	8
Song	2019	USA	Elisa	Serum	36	DF: 9, MM: 14	Elisa Kits	8

Table S5. Newcastle–Ottawa Scale (NOS) score of included studies.

Included studies	Year	Is the definition adequate?	Representativeness of the cases	Selection of controls	Definition of controls	Comparability of both groups	Ascertainment of diagnosis	Same ascertainment method for both groups	Nonresponse rate	Total scores
Zhou	2005	☆	-	☆	☆	☆☆	☆	☆	☆	8
Kadkol	2006	☆	-	☆	☆	☆	☆	☆	☆	7
Packer	2006	☆	-	☆	☆	☆	☆	☆	☆	7
Reiniger	2006	☆	-	☆	☆	☆	☆	☆	☆	7
Barak	2007	☆	-	☆	☆	☆	☆	☆	☆	7
Soikkeli	2007	☆	-	☆	☆	☆	☆	☆	☆	7
Haritoglou	2009	☆	-	☆	☆	☆	☆	☆	☆	7
Elias	2010	☆	-	☆	☆	☆	☆	☆	☆	7
Maier	2011	☆	☆	☆	☆	☆☆	☆	☆	☆	9
Zhang	2012	☆	☆	☆	☆	☆☆	☆	☆	☆	9
Kiss	2015	☆	-	☆	☆	☆☆	☆	☆	☆	8
Song	2019	☆	☆	☆	☆	☆	☆	☆	☆	8

Table S6. Publications bias.

Comparisons	P value (Egger's test)
Nevi vs. PM	0.101
Nevi vs. MM	0.544
PM vs. MM	0.693
Health persons vs. MM patients	0.069
PM patients vs. MM patients	-
DF patients vs. MM patients	0.645

Table S7. The correlations of SPP1 with clinillcopathological features of melanoma patients.

	n	SPP1 expression		p-value
		low	high	
Gender				
Male	15	7	8	1.000
Female	4	2	2	
Age (years old)				
<60	10	5	5	1.000
≥60	9	4	5	
Diameter of lesion (cm)				
<10	3	2	1	0.582
≥10	16	7	9	
Breslow thickness (mm)				
<4	7	6	1	**0.020**
≥4	12	3	9	
Clark classification				
I-II	5	2	3	1.000
III-V	14	7	7	
Satellite lesions				
Absent	16	8	8	1.000
Present	3	1	2	
Metastasis				
Absent	12	5	7	0.650
Present	7	4	3	
Ulcers				
Absent	13	6	7	1.000
Present	6	3	3	

Table S8. The cox proportional hazard regression analyses for overall survival in melanoma patients.

Variables	Univariable analysis		multivariable analysis	
	HR (95% CI)	P value	HR (95% CI)	P value
Gender (female vs. male)	0.537 (0.067-4.308)	0.559	0.273 (0.002-35.673)	0.602
Age (≥60 vs. ＜60)	2.144 (0.601-7.644)	0.240	1.172 (0.170-8.096)	0.872
tumor diameter(≥10cm vs. ＜10cm)	2.167 (0.266-17.644)	0.470	0.066 (0.000-14.720)	0.325
Breslow thickness(≥4mm vs. ＜4mm)	1.125 (0.280-4.514)	0.868	0.122 (0.009-1.656)	0.114
Clark classification(≥3 vs. ＜3)	1.236 (0.306-4.989)	0.766	1.068 (0.069-16.453)	0.962
Satellite lesions (presence vs. absence)	1.783 (0.359-8.858)	0.479	2.475 (0.155-39.501)	0.521
Metastasis (presence vs. absence)	0.996 (0.241-4.107)	0.995	4.859 (0.353-66.954)	0.238
Ulcers (presence vs. absence)	0.453 (0.096-2.144)	0.318	0.233 (0.015-3.664)	0.300
SPP1 expression (high vs. low)	4.791 (1.004-22.85)	**0.049**	50.699 (1.515-1697.050)	**0.028**

HR : hazard risk; CI: confidence interval.

Table S9. Transcriptional factor of SPP1 in recent years.

Studies	Year	Country	Detect methods	Transcriptional factor	Cell lines	PMID
Bidder	2002	USA	Luciferase assay	AP-1, USF	vascular smooth muscle cells	12200434
Renault	2005	France	Luciferase assay	NF-κB, AP-1	arterial smooth muscle cells	15557322
Samant	2007	USA	Gel shift and ChIP	NF-κB	MDA-MB-435 cells	17227585
Sharma	2010	India	Gel shift and ChIP assay	AP-1	HeLa cells	20609221
Lee	2010	Korea	Luciferase assay	Runx2, MEF	rat osteoblast cells	20498758
Zhao	2011	China	Chromosome conformation capture technology	NF-κB, AP-1	murine macrophages	21257959
Sowa	2013	Germany	Luciferase assay	Runx2, Vdr	HeLa and HEK293 cells	23644099
Cheng	2013	China	CHIP assay	RUNX3	AGS and SCM-1 cells	23774402
Lyle	2014	Japan	ChIP assays	NF-κB, AP-1	vascular smooth muscle cells	24247243
Wang	2016	China	ChIP and luciferase assay	Gfi1	3T3-L1 cells	27283242
Zhang	2016	China	CHIP assay	Smad4	Huh-7 and A549 cells	26584547
Zhang	2019	China	EMSA and ChIP assay	Foxo1	macrophages	31096186
Takami	2019	Japan	Gel shift and ChIP assays	Sp1	SW480 cells	17689681

ChIP: chromatin immunoprecipitations; EMSA: electrophoretic mobility shift assay
